# Digital Outpatient Care for Patients With Type 1 Diabetes (DigiDiaS): Pragmatic Observational Pre-Post Study

**DOI:** 10.2196/94782

**Published:** 2026-07-13

**Authors:** Ingeborg Spildo, Heidi Holmen, Annesofie Lunde Jensen, Milada Hagen, Tone Singstad, Jacob Andreas Winther, Eirik Arsand, Astrid Torbjørnsen

**Affiliations:** 1 Department of Nursing and Health Promotion Faculty of Health Sciences OsloMet – Oslo Metropolitan University Oslo, Oslo Norway; 2 Department of Clinical Medicine Aarhus University Aarhus, Central Jutland Denmark; 3 Steno Diabetes Centers Aarhus Aarhus, Central Jutland Denmark; 4 Department of Endocrinology Akershus University Hospital Lørenskog, Akershus Norway; 5 Department of Computer Science University of Tromsø, The Arctic University of Norway Tromsø, Troms Norway

**Keywords:** delivery of health care, diabetes mellitus type 1, distance counselling, patient reported outcome measures, remote consultation, telemedicine, telenursing

## Abstract

**Background:**

Patient-reported outcomes in digital health solutions can offer patients with type 1 diabetes an opportunity to voice their needs in outpatient care, enabling clinicians to tailor support. Evidence on long-term health impact and routine integration of such digital solutions outside controlled settings is limited.

**Objective:**

This study aimed to compare a flexible digital supplement to outpatient care for type 1 diabetes (DigiDiaS) with usual care over 1 year, with self-management as the primary outcome and glycemic control and well-being as secondary outcomes.

**Methods:**

This longitudinal real-world observational pre-post study was conducted at the Endocrinology Department of Akershus University Hospital in Norway. Adults with type 1 diabetes were recruited consecutively from October 2022 to October 2023 and could choose either the digital mobile health (mHealth)–based outpatient care model—“DigiDiaS” care—or usual care. DigiDiaS care is delivered via a smartphone app and includes a messaging service; patient-reported outcome–based preconsultation questionnaires; video, telephone, and in-person consultations; and an individually tailored information section. Outcome data comprised self-reported measures, clinical data extracted from electronic medical records, the national diabetes registry, and the digital platform. Data were collected at baseline and at the 1-year follow-up. Change in self-management (Patient Activation Measure short version [PAM-13]; primary outcome) and the secondary outcomes well-being (Five Well-Being Index [WHO-5]) and glycemic control (glycated hemoglobin [HbA_1c_]) were analyzed using a generalized linear model. Utilization of the digital solution and health care services was also explored.

**Results:**

We included 237 patients, with 185 (78%) opting for DigiDiaS care and 52 (22%) for usual care. At the follow-up, most participants (145/178, 81%) in the DigiDiaS care group used the digital solution. The messaging service was the most utilized feature with 592 messages sent collectively (median 1, min-max 0-57). There were no statistically significant between-group differences in change from baseline to follow-up in self-management (mean difference 1.18, 95% CI –5.2 to 7.6; *P*=.72), glycemic control (HbA_1c_ mean difference –2.1, 95% CI –6.6 to 2.4; *P*=.36), or well-being (mean difference 1.9, 95% CI –3.1 to 6.9; *P*=.46). Health care utilization did not differ between the groups, including participation in one or more individual consultations during the 1-year follow-up (DigiDiaS care: 159/178, 89%; usual care: 42/50, 84%; *P*=.30) or appointment cancellations (DigiDiaS care: 64/91, 70%; usual care: 13/18, 72%; *P*=.20).

**Conclusions:**

This real-world pragmatic observational comparison under routine conditions demonstrates that reorganizing outpatient care for patients with type 1 diabetes into a flexible digital model, DigiDiaS care, did not result in statistically significant between-group differences in health outcomes, including self-management, glycemic control, and well-being, compared with usual care. Over 80% of participants in DigiDiaS care utilized the digital solution, with patient-initiated asynchronous messaging as the most frequently used feature.

**International Registered Report Identifier (IRRID):**

RR2-10.2196/52766

## Introduction

Living with type 1 diabetes requires individuals to continuously self-manage their condition to prevent both short- and long-term complications, which can profoundly affect daily life [1-3]. This ongoing need for self-management not only plays a central role in disease control but may also impose a substantial mental and emotional burden [4,5]. Support from health care professionals may therefore be essential, providing medical guidance and emotional support to help patients navigate the challenges of self-managing their condition and maintaining well-being [3,6].

Follow-up care from health care services should be tailored to patients’ individual needs and flexible enough to fit seamlessly into patients’ daily routines [7,8]. Patients with long-term conditions can contribute with insight into when and how support is needed [9], and patient-reported outcome measures (PROMs) can help capture these insights [10,11]. PROMs can facilitate interactions between health care professionals and patients by supporting shared reflection, encouraging patients to articulate concerns and priorities, and enabling clinicians to address specific symptoms and problems that patients experience [12]. Digital solutions can be designed to collect and manage PROM data, and integrate these PROM data into clinical workflows, enabling personalized, patient-centered care that aligns with patients’ lives and preferences [13,14].

Although digital solutions that integrate PROMs into outpatient care appear promising, a recent scoping review identified only 4 PROMs implemented in diabetes outpatient care, either in waiting rooms or at home before consultations [15]. Among these, the Danish “DiabetesFlex” program is one of the most comprehensive, and studies provide insights into potential benefits of PROM-based digital care [16,17]. A “DiabetesFlex” randomized controlled trial reported improved diabetes-related well-being, patients’ preference for video consultations over face-to-face visits, and reduced nonattendance after 15 months of follow-up [17]. Qualitative interviews with health care professionals from DiabetesFlex also suggested strengthened patient-clinician partnerships and increased patient self-reflection [16,18]. However, some participants did not wish to continue with DiabetesFlex after the study period [17]. Nonuse of digital PROM solutions varies across contexts and is often related to technical barriers, low perceived benefit, or participant burden [19]. Together, findings from DiabetesFlex and nonuse of digital PROM underline the need to study digital solutions outside controlled research settings to understand potential effects on health outcomes and the extent to which such solutions are utilized in routine clinical practice over time.

Building on experience from DiabetesFlex, the endocrinology outpatient clinic at Akershus University Hospital introduced a voluntary remote digital outpatient care platform for patients with type 1 diabetes, “DigiDiaS” in November 2021 [20]. We previously described demographic, clinical, and diabetes-specific characteristics of patients who opted for DigiDiaS care compared with those who opted for usual care [21]. Patients who chose DigiDiaS care tended to have shorter diabetes duration, were more likely to use an insulin pump (rather than an insulin pen), and reported lower well-being than patients who chose usual care. Qualitative interviews with clinicians and patients using DigiDiaS care at the Endocrinology Department at Akershus University Hospital suggest that health care professionals’ acceptance and use of DigiDiaS care influence patients’ willingness to adopt and use the platform [22]. However, the longer-term differences between DigiDiaS care and usual care on patient outcomes and patterns of platform use remain unclear.

This study aimed to compare a flexible digital supplement to outpatient care for type 1 diabetes (DigiDiaS) with usual care over 1-year, with self-management as the primary outcome and glycemic control and well-being as secondary outcomes.

## Methods

### Study Design

This study used a real-world quasi-experimental, pragmatic, nonrandomized, longitudinal observational pre-post design to examine changes in health-related outcomes from baseline to a 1-year follow-up between patients receiving DigiDiaS care compared with usual care. Data were collected as part of a multimethod prospective observational study of the introduction and integration of a digital outpatient care approach for patients with type 1 diabetes and health care professionals, as described in the study protocol [20]. The main deviation from the study protocol was the use of a generalized linear model (GLM) rather than ANOVA, as a GLM approach allowed for covariates and more appropriate handling of repeated measures and missing data. Other minor deviations are detailed in Multimedia Appendix 1. The study is reported in accordance with the STROBE (Strengthening the Reporting of Observational Studies in Epidemiology) reporting guideline [23].

### Setting and Participants

The study was conducted at the endocrinology outpatient clinic of Akershus University Hospital in southeast Norway.

Eligible participants were aged 18 years or older, diagnosed with type 1 diabetes, receiving treatment and care at the Akershus University Hospital endocrinology outpatient clinic, and able to read Norwegian. Individuals with type 2 diabetes, gestational diabetes, those who could not read or understand Norwegian, and those with cognitive or communication difficulties likely to preclude informed consent or questionnaire completion were not eligible for enrollment in the study. No formal cognitive screening instrument or score was used; this exclusion criterion was applied pragmatically. Participants who became pregnant, moved, or otherwise received diabetes follow-up care from other specialist services during the study period were also excluded. Both users and nonusers of DigiDiaS care were eligible for inclusion.

### Recruitment

Recruitment took place from October 2022 to October 2023, and participants were followed for 1-year from the date of consent. Outcome data collection was completed in November 2024. We used consecutive sampling for recruitment and allowed participants to choose DigiDiaS care or to continue with usual care. Eligible patients were invited to participate in the study during appointments with a diabetes specialist nurse or endocrinologist. Patients received written and oral information about the study from their diabetes specialist nurse or endocrinologist and were asked whether a researcher (author IS) could contact them for further information and answer any questions. Those who agreed were contacted by phone for further information and then sent a link to a digital consent form by SMS text message. A paper consent form was available on request. If a patient expressed interest in participating during the telephone call but did not complete the digital consent form, 1 SMS text message reminder was sent.

### Diabetes Outpatient Care

#### Usual Care

Usual care for adults with type 1 diabetes at the endocrinology outpatient clinic involves routine, calendar-based follow-up appointments in accordance with national diabetes guidelines [24]. In Norway, patients with type 1 diabetes are offered prescheduled, calendar-based follow-up care in specialist outpatient settings, delivered by interdisciplinary teams consisting of endocrinologists, diabetes specialist nurses, and clinical nutritionists, according to individual need. Usual care includes a comprehensive annual checkup and additional routine checkups between annual evaluations, as needed [24]. Patients with recent diabetes diagnosis, comorbidities, late complications, or suboptimal glycemic control are offered more frequent consultations based on clinical need.

Diabetes care in Norway is subject to a small annual out-of-pocket limit of NOK 3278 (approximately US $320), which covers outpatient care, necessary medicines, and diabetes management equipment. Once this limit is reached, all additional expenses are fully covered by the welfare state.

#### DigiDiaS Care

DigiDiaS care is a flexible digital supplement designed to support interactions between patients and health care professionals during diabetes outpatient care, with tailoring to individual needs [20,21]. DigiDiaS care is delivered to patients through a free app available in the App Store and Google Play. Patients require an invitation from health care professionals to download the app and sign in on their own smartphones.

Dignio Connected Care delivers the solution to patients through the native app “MyDignio” and to health care professionals through the web app “DignioPrevent,” accessible via a web browser and linked directly from the electronic health record system. Dignio Connected Care is CE (Communauté Européenne)–certified [25]. Multimedia Appendix 2 presents illustrative patient-facing screenshots, including an example item from the preconsultation patient-reported outcome (PRO)–based questionnaire and the asynchronous messaging feature. All patients opting for DigiDiaS care received detailed information about “MyDignio,” with particular focus on the messaging service [20,22]. Assistance with downloading and accessing the platform was offered when needed.

**Figure 1:**
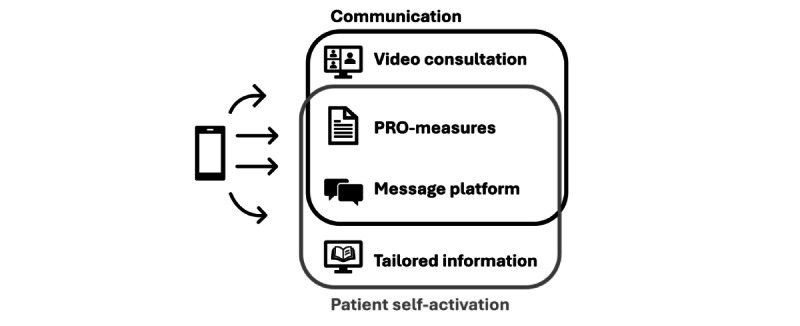
Features of DigiDiaS care. PRO: patient-reported outcome.

The 4 core features of DigiDiaS care are intended to support communication and patient self-activation through interaction with health care professionals (Figure 1). First, before scheduled consultations, patients received a PRO-based preconsultation questionnaire covering well-being, diabetes-specific outcomes, and the topics they wished to discuss. In total, 2 PRO-based questionnaires were developed: one for the extended annual checkup with a diabetes specialist nurse or endocrinologist and one for routine checkup with a diabetes specialist nurse. Questionnaire content was adapted from the Danish DiabetesFlex program to the Norwegian context [17,20,26]. Health care professionals handle responses and may rate them using a traffic-light model (green, yellow, and red) for guidance. The traffic-light model highlights areas of concern, including alerts when questionnaires remain unanswered. Second, an asynchronous messaging service enables communication between patients and the health care service outside planned appointments. When needed, the diabetes specialist nurse handling the messages may schedule a consultation. Third, for routine checkups with diabetes specialist nurses, patients can choose between in-person, video, or telephone consultations. Video consultations are conducted through a separate system (“Whereby”) [27]. Finally, clinicians can provide an individually tailored information section, including topic templates, access to an e-learning course, and personalized free-text content. Patients receive notifications about new activities via push notifications or SMS text messages.

### Data Collection

Data were collected immediately after signed consent at baseline, and at the 1-year follow-up from 3 sources: self-reported data, information extracted from the Distributed Information and Patient Data System in Hospitals electronic health record system, and data from the National Quality Registry for Diabetes (“The Norwegian Organization for Quality Improvement of Laboratory Examinations” [NOKLUS]). At follow-up, usage data recorded in the digital care platform were extracted from DignioPrevent, as outlined in the protocol [20].

All data were managed, stored, and analyzed within the services for sensitive data (TSD), in compliance with strict data handling guidelines [28]. Self-reported data were collected via the secure web-based survey solution “Nettskjema” [29], which transfers encrypted data directly to TSD, ensuring secure and confidential data handling. Self-reported data recorded on paper were manually entered into Nettskjema by the first author (IS) and thereby transferred to TSD, while the original paper versions were stored in a locked safe. Data from participants’ electronic health records, NOKLUS, and DignioPrevent were manually extracted and entered into Microsoft Excel worksheets and stored in TSD. Data extraction was performed by the first author (IS) and controlled by a coresearcher.

Participants could complete the baseline outcome questionnaire immediately after providing digital consent, via a secure link sent by SMS text messages or email, within 2 weeks, or on paper returned by post in a prepaid envelope, according to preference. At the 1-year follow‑up, the outcome questionnaire was likewise available via a secure link (SMS text message or email) or as a paper version sent by post with a prepaid return envelope. Follow‑up questionnaires were sent to all participants, irrespective of whether they had completed the baseline questionnaire. For unreturned questionnaires, an automatic reminder was sent 5 days after initial contact. If the participant remained unresponsive, the first author (IS) contacted them by telephone, followed by an SMS text message, 2 additional automatic reminders, and finally, the questionnaire was sent by post to participants who still had not responded.

### Measures

#### Demographics and Clinical Variables

Sociodemographic and clinical measures, including gender, age, education, diabetes duration, and BMI, were collected from the electronic health record system at baseline. Information on late complications from diabetes, diabetic ketoacidosis, hypoglycemia, and comorbidities was also retrieved from the electronic health records and the NOKLUS diabetes registry at baseline. Clinical and diabetes-specific variables, such as insulin delivery method (pump/pen), blood glucose monitoring method (sensor/glucometer), glycated hemoglobin (HbA_1c_), time in range, blood pressure, and low-density lipoprotein cholesterol, were collected at baseline and at 1-year follow-up from the NOKLUS diabetes registry. Measures and blood samples were obtained within 3 months before or after the 1-year follow-up date. For detailed variable descriptions on categories, how they were merged for analysis, interpretations, estimands, and data collection points, see Multimedia Appendix 3.

#### Health Care Utilization

Health care utilization during the 1-year follow-up was assessed using data obtained from the electronic health record system. This included the number of individual consultations with a diabetes specialist nurse, endocrinologist, or clinical nutritionist, as well as the mode of consultation, whether in person, by telephone, or via video. Group education sessions and courses were also included, such as a diabetes education course, carbohydrate counting course, and insulin pump initiation or switching training sessions. In addition, we collected data on appointment changes (initiated by the patient or the clinic), nonattendance, and cancellations made less than 24 hours before a scheduled consultation or group education session. Finally, information on hospital admissions for ketoacidosis and hypoglycemia during the follow-up period was retrieved from the electronic health records system based on diagnosis code.

#### Usage Data From the Digital Solution

We extracted data on patients’ use of the digital solution from DignioPrevent, including the number of messages sent from participants to the clinic, the number of messages sent from the clinic to participants, the number of distributed PRO-based questionnaires, the number of completed PRO-based questionnaires, and whether the individually tailored information section was in use. In addition, we recorded the date of first login and any reconsent for app use. If the MyDignio app is reinstalled or has not been used for more than 90 days, or if the participant changes mobile phone, the user must reconsent when logging in. A recorded reconsent confirms that the participant logged in and used the app and was therefore counted as app use. Platform use was defined both as “access events” (secure login or reconsent) and as “active clinical use” (patient-initiated messages and/or completion of a PRO questionnaire). All functions are delivered within the same app, but passive viewing of tailored information is not logged.

#### Primary Outcome

The primary outcome was the between-group difference in change in self-management from baseline to 1-year follow-up, measured with the Patient Activation Measure short version (PAM-13) [30].

PAM-13 is a generic questionnaire, developed and validated by Hibbard et al [30,31], suitable for individuals with chronic conditions. The PAM-13 is validated in Norwegian for patients with a range of chronic diseases [32,33], and has been used to compare patient education in various diagnoses [34,35], including type 2 diabetes [36]. PAM-13 has previously been used to evaluate digital interventions across diverse settings and contexts, including diabetes [37]. Cronbach α for self-management at baseline in our data was 0.894, indicating high internal consistency [21]. For details on the scale, domains, interpretations, estimands, and data collection points, see Multimedia Appendix 3.

#### Secondary Outcomes

##### Well-Being

Psychological well-being and quality of life were measured using the Five Well-Being Index (WHO-5), a generic tool developed by the World Health Organization in 1998 [38]. The WHO-5 effectively measures clinical concepts and outcomes in a reliable and meaningful way. The questionnaire has been translated into over 30 languages, including Norwegian [39], and is validated for use in type 1 diabetes [40,41]. Cronbach α for well-being in our data at baseline was 0.826, showing good internal consistency [21]. For details on scale, domains, interpretations, estimands, and data collection point, see Multimedia Appendix 3.

##### Diabetes Distress

Diabetes distress was compared using the disease-specific questionnaire “Problem Areas in Diabetes” (PAID-20), developed by Polonsky et al [42], and translated and validated in Norwegian by Graue et al [43]. The PAID-20 questionnaire has previously been used in a feasibility study of a digital intervention involving 69 participants at a different Norwegian diabetes clinic, where PRO questionnaires were administered in the waiting room prior to consultation [44]. In our study, the Cronbach α for diabetes distress at baseline was 0.939, reflecting high internal consistency [21]. For details on the scale, domains, interpretations, estimands, and data collection points, see Multimedia Appendix 3.

##### Health Literacy

Health literacy and the ability to make informed health choices were compared using the short version of the European Health Literacy Survey Questionnaire (HLS19-Q12) [45]. The HLS19-Q12 has been translated into multiple languages and used in 19 European countries [46]. It has been translated and validated in Norwegian and applied to both a large general population and patients with type 2 diabetes [45,47,48]. Cronbach α for health literacy at baseline in our study was 0.871, indicating good internal consistency [21]. For details on the scale, domains, interpretations, estimands, and data collection points, see Multimedia Appendix 3.

##### Digital Health Literacy

Digital health literacy includes a variety of knowledge and skills required to use electronic tools in handling one’s own health and illness [48]. This includes using digital tools and communicating with health care professionals during follow-up care. The questionnaire “Health Literacy Survey 2019-2021, digital skills, Norwegian part“ (HLS19-DHC-NO_Norwegian) addresses the use of digital health care [49]. HLS19-DHC-NO was developed in Norway by a Norwegian research group and validated for adolescents and young adults in the general population aged 16-25 years [49]. The reliability of the HLS19-DHC-NO scale in this study was confirmed by a Cronbach α of 0.930, indicating excellent internal consistency among the items. Since the population for which the instrument was validated is not comparable to the participants in this study, and there is no established interpretation of what different scores represent, we used a chi-square test to compare the proportions of the highest and lowest scores and reported missing data for each item. Digital health literacy was added as an outcome during the study to capture digital engagement and was therefore assessed only at the 1‑year follow‑up. For details on the scale, domains, interpretations, estimands, and data collection point, see Multimedia Appendix 3.

##### Experience of Involvement

The patients’ experience of involvement in treatment and patient cooperation with health care professionals was measured using 5-items. The items were adapted from Danish [50], translated into Norwegian (author IS), back-translated by a native Danish speaker with 10 years’ residence in Norway, and finalized by consensus (authors ALJ, AT, and IS) for linguistic and cultural validity. The Danish version of the experience of involvement have been validated and English translation is available [51]. For details on the scale, domains, interpretations, estimands, and data collection points, see Multimedia Appendix 3.

### Sample Size

The power calculation was conducted for the primary outcome, self-management, measured with the PAM-13 questionnaire, and is further described in the study protocol [20]. We used unpublished PAM-13 data from 2539 Norwegian patients with various chronic diseases [52]. We expected a small change in self-management in the DigiDiaS care group, corresponding to a score change of 4 in the intervention group and 3.8 in the control group. Thus, we assumed a raw mean score of 44.4 (SD 5.6) and 44.6 (SD 5.3) for DigiDiaS care and usual care, respectively, after 6 months (within a 12-month recruitment period from baseline), and an absolute between-group difference of 0.2 at 1-year when assessed using the PAM-13 raw score. Based on these assumptions, the minimum required sample size was n=32 in order to detect the above-defined difference as statistically significant (*P*<.05). We further anticipated a dropout of at least 20% for the longitudinal part of the study. We therefore included 52 participants in the usual care group at baseline.

### Ethical Considerations

This research project involves human participants and was reported to the Norwegian Agency for Shared Services in Education and Research under reference number 456954 and received approval from the local Data Protection Officer at Akershus University Hospital (reference number 2022_125). The project was reviewed by the Regional Committees for Medical and Health Research Ethics (reference number 407539), who determined that it fell outside their jurisdiction concerning health research, and classified it as a quality improvement initiative [53].

Participants were provided with both oral and written information before providing their digital consent to participate in this study. Contact information with phone numbers and email addresses for authors IS and AT was included in all written materials distributed to participants. Digital consent forms, along with data from electronic health records and self-reported questionnaires, were stored and managed within the TSD. Participants submitted their Social Security numbers on the consent form but were assigned unique IDs to ensure deidentification. The key linking IDs to personal data was kept in a separate file within TSD. Participants did not receive any compensation for their involvement in the study.

### Statistical Analyses

#### Overview

Categorical variables are presented as counts and percentages, while continuous variables are reported as medians with minimum and maximum values, given the unequal group sizes. Data on health care utilization are presented as sums and, when relevant, as percentages of those sums.

To explore comparability of group characteristics at baseline, nonparametric Mann-Whitney *U* tests were applied for continuous variables and chi-square tests for categorical variables. Between-group differences in change (DigiDiaS care vs usual care) were analyzed using a GLM with repeated‑measures linear mixed‑effects. Models were fitted with fixed effects for time (baseline, 1-year), care model (DigiDiaS vs usual care), and their interaction (time × care model), and with a participant‑level random intercept. We did not force any parametric structure on the data and used an unstructured covariance matrix. We fitted models adjusted for prespecified baseline covariates considered potential confounders (diabetes duration, insulin delivery method, and WHO-5 well-being score), which were previously identified as baseline differences between DigiDiaS care and usual care in a multivariate logistic regression [21]. Analyses adjusted for age, gender, and baseline HbA_1c_ are presented in Multimedia Appendix 4, and an unadjusted model is presented in Multimedia Appendix 5. Results are presented as estimated means with corresponding 95% CIs. Differences in change are reported as the estimated between-group mean difference (MD), with corresponding 95% CIs and *P* values. Changes in categorical variables were calculated as specified in Multimedia Appendix 3 for electronic health record data and for self-reported data. Health care utilization was compared between the 2 groups using a Mann-Whitney *U* test.

All data management and analyses were conducted using Microsoft Excel worksheets (Office Professional Plus 2016) and SPSS (version 29.0.0.0; IBM Corp) for the initial bivariate statistical analyses. GLMs were subsequently performed in Stata (version 17; Stata Corp). All tests were 2-sided. The study was considered exploratory, so no correction for multiple testing was applied, and *P*-values <.05 were considered statistically significant.

#### Missing Data

Responses such as “don’t know” or “not applicable” in the self-reported questionnaires were treated as missing values. The amount of missing data, or the number of participants included, is reported for each variable. This was necessary because the number of participants varied between baseline and 1-year follow-up due to dropout and differences in the availability of data from self-reported questionnaires and electronic health records. No missing data were imputed.

We conducted a sensitivity analysis on the missing data of the primary outcome. Two extreme‑case scenarios were applied to missing follow‑up values for the primary outcome of self-management (PAM‑13): worst‑case, replacing all missing follow‑up scores with the minimum possible value, and best‑case, replacing all missing follow‑up scores with the maximum possible value. The adjusted primary model was adjusted for the same baseline covariates: diabetes duration, insulin delivery method, and WHO-5 well-being score as in the original analysis presented in Multimedia Appendix 6.

#### As-Treated Analysis

The primary analyses were conducted according to the initial group choice, classifying participants as DigiDiaS care or usual care according to their choice of care model at baseline. For those opting for DigiDiaS, assignment was operationalized as confirmed if a first login to the MyDignio app occurred within 90 days of baseline study consent, based on login times‑stamped in DignioPrevent. Group status was recorded at the point of medical records data collection.

To account for participants who switched from usual care to DigiDiaS care during the study period, an as‑treated classification reallocated participants by status at 1 year, defining DigiDiaS as a first login within 90 days before the follow‑up date. Results of the as-treated analyses are presented in Multimedia Appendices 7-10.

#### Responders and Nonresponders

To assess potential responder bias, responders (baseline and follow‑up) were compared with nonresponders (baseline only) on selected characteristics for the self‑reported outcome questionnaire, within the DigiDiaS care and usual care groups. Responders and nonresponders were comparable on most characteristics in DigiDiaS care and usual care; however, in the DigiDiaS care group, responders had a longer diabetes duration than nonresponders (median 19, min-max 0-49 years vs median 13.5, min-max 0-59 years; *P*=.04). See further details in Multimedia Appendix 11.

## Results

### Overview

**Figure 2:**
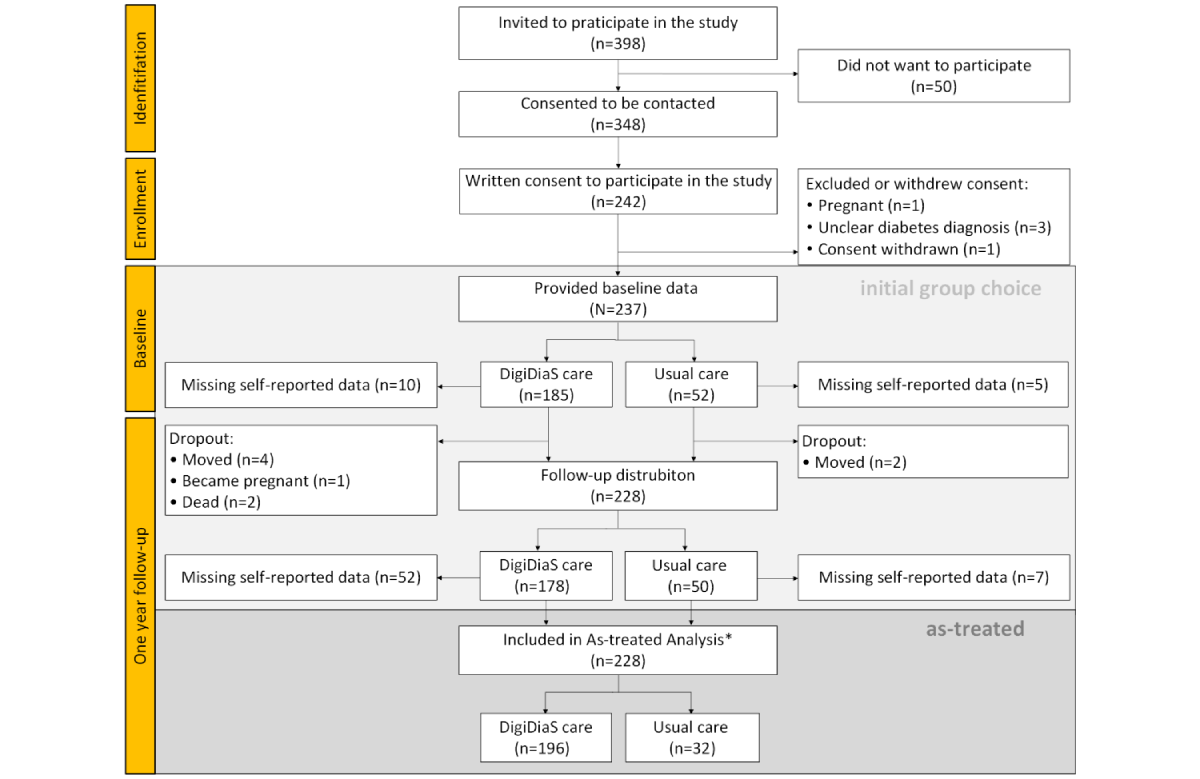
Flowchart of recruitment, distribution, group changes, and missing self-reported data in initial group choice and as-treated analysis. Dropout numbers are identical in both the as-treated and initial group choice analyses. To the best of our knowledge, none of the deaths were related to participation in this study. *A total of 18 participants originally in usual care moved to DigiDiaS care during the 1-year follow-up. As-treated counts are based on updated group allocation at 1 year. The distribution of missing self-reported data and dropout is the same in the as-treated analysis as in the initial group choice analysis.

During the recruitment period, 237 patients consented to participate in the study. Most (n=185, 78.1%) chose DigiDiaS care, while the remaining participants (n=52, 21.9%) opted for usual care (Figure 2). At baseline, self-reported data were missing for 21 (11.4%) participants in the DigiDiaS care group and 5 (9.6%) participants in the usual care group. Over the 1-year follow-up, 9 (3.8%) participants dropped out of the study: 7 (3.8%) from the DigiDiaS care group and 2 (3.8%) from the usual care group. Reasons for dropout included relocation outside the hospital coverage area, receiving outpatient care elsewhere, pregnancy, or death. At follow-up, the DigiDiaS care group included 178 participants, and the usual care group included 50 participants. Self-reported data on the primary outcome were missing for 52 (29.2%) participants in the DigiDiaS care group and 7 (14%) participants in the usual care group. During the study period, 18 (7.9%) participants switched from the usual care group to DigiDiaS care. In the as-treated analysis, 196 (82.7%) participants were receiving DigiDiaS care, and 32 (13.5%) were receiving usual care at the end of follow-up.

### Baseline Characteristics

Despite the lack of randomization, the DigiDiaS care and usual care groups were comparable across key baseline characteristics, except for gender, age, diabetes duration, insulin delivery method, and HbA_1c_ (Table 1). There was a statistically significantly larger proportion of women in the DigiDiaS care group (n=91, 49.2%) than in the usual care group (n=17, 32.7%; *P*=.04). Participants in the DigiDiaS care group were statistically significantly younger, with a median age of 47 (min-max 19-79) years compared with 59 (min-max 23-81) years in the usual care group (*P*<.001). In addition, participants in the DigiDiaS care group had a median diabetes duration approximately 10 years shorter (DigiDiaS care: median 19, min-max 0-51 years; usual care: median 29, min-max 3-58 years; *P*<.001). Insulin pump usage was statistically significantly more common in the DigiDiaS care group (n=74, 40%) than in the usual care group (n=13, 23.1%; *P*=.03). The DigiDiaS care group also had a statistically significantly higher median HbA_1c_ of 59 (min-max 35-117) mmol/mol compared with 52.5 (min-max 41-90) mmol/mol in the usual care group (*P*=.004).

Digital health literacy was assessed only at follow-up, and between-group proportions of the highest and lowest scores were compared. For item 5, “Using a mobile phone or tablet to record results from measurements you take yourself (eg, blood pressure),” participants in usual care were more likely to report low confidence than those in DigiDiaS care (*P*=.02; see Multimedia Appendix 12). No statistically significant differences were observed for the remaining items between DigiDiaS care and usual care.

**Table 1:** Demographic and clinical variables at baseline [21]^a^.

		Total (n=237)	DigiDiaS care (n=185)	Usual care (n=52)	*P* value		
Gender (woman), n (%)		108 (45.6)	91 (49.2)	17 (32.7)	.04		
Age (years), median (min-max)		49 (19-81)	48 (19-79)	59 (23-81)	<.001		
Education, n (%)						.41	
	Education up to upper secondary school or vocational school (13 years)	87 (36.7)	70 (37.8)	17 (32.7)			
	University/college education up to 4 years	49 (20.7)	41 (22.2)	8 (15.4)			
	University/college education more than 4 years	32 (13.5)	23 (12.4)	9 (17.3)			
	Missing	69 (29.1)	51 (27.6)	18 (34.6)			
Diabetes duration (years), median (min-max)		20 (0-59)	18 (0-59)	28 (2-58)	<.001		
Blood glucose monitoring: continuous glucose monitoring, n (%)		225 (94.9)	178 (96.2)	47 (90.4)	.05		
	Missing	1 (0.4)	1 (0.5)	0			
Insulin delivery: pump, n (%)		86 (36.3)	74 (40.0)	12 (23.1)	.02		
	Missing	1 (0.4)	1 (0.5)	0			
HbA_1c_^b^ in mmol/mol, median (min-max)		57 (33-124)	58.5 (35-124)	52 (33-90)	.004		
	Missing, n (%)	2 (0.8)	1 (0.5)	1 (1.9)			
HbA_1c_ above 75 mmol/mol, n (%)		36 (15.2)	32 (17.3)	4 (7.7)	N/A^c^		
Late complications from diabetes, n (%)						.76	
	None^d^	136 (57.4)	107 (57.8)	29 (55.8)			
	1^d^	63 (26.6)	51 (27.6)	12 (23.1)			
	≥2^d^	38 (16.0)	27 (16.6)	11 (21.1)			
Comorbidities, n (%)						.62	
	None^d^	96 (40.5)	77 (41.6)	19 (36.5)			
	1^d^	77 (32.5)	59 (31.9)	18 (34.6)			
	≥2^d^	37 (16.0)	27 (14.6)	10 (19.2)			
	Missing	26 (11.0)	21 (11.9)	5 (9.6)			
BMI (kg/m^2^), median (min-max)		26.5 (16.9-43.9)	26.4 (16.9-43.9)	26.8 (19.4-42.7)	.71		
	Missing, n (%)	4 (1.7)	3 (1.6)	1 (1.9)			

^a^The number of respondents for self-reported data may vary due to incomplete questionnaires and dropout from baseline to follow-up.

^b^HbA_1c_: glycated hemoglobin.

^c^N/A: not applicable.

^d^Merged into categories for analysis.

### Use of DigiDiaS care

Over 80% (145/178, 81.5%) of participants in the DigiDiaS care group used the app during the study period, engaging with one or more features such as the messaging service, completing a PRO-based questionnaire, or reconsenting to app use. Fewer than 1 in 5 (33/178, 18.5%) participants did not use the app at all during the study period (Table 2).

**Figure 3:**
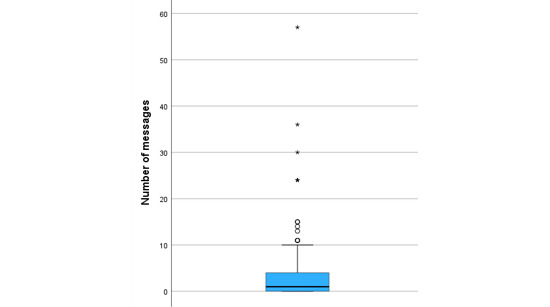
Boxplot of the number of messages sent from participants to the health care service. Circles (o): mild outliers, which fall between 1.5 and 3 times the IQR from the box. Stars (*): extreme outliers, which fall more than 3 times the IQR from the box.

Given the individualized nature of DigiDiaS care, app use varied substantially between participants. The PRO-based questionnaire was distributed to 123 (69.1%) of the 178 participants in DigiDiaS care, with each participant receiving it one or more times (Table 2). Overall, 58% (n=94) of the 162 distributed questionnaires were completed. The most frequently used feature was the messaging service: 109 (61.2%) participants sent messages to the health care service with 592 messages in total (median 1, min-max 0-57), resulting in both mild and extreme outliers illustrated in the boxplot (Figure 3). The endocrinology outpatient clinic sent general information messages to app users on 2 occasions during the study period; 61 (34.3%) participants received a message on the first occasion, and 112 (63%) participants received messages on both occasions. For further details on the use of DigiDiaS care features, see Table 2.

**Table 2:** Use of the features of the DigiDiaS care.

DigiDiaS care		Value (n=178)
Overall patient use, n (%)		
	One or more messages sent	109 (61.2)
	One or more PRO questionnaires received	123 (69.1)
	One or more PRO questionnaires completed	87 (48.8)
	Nonusers	33 (18.5)
PRO^a^-based questionnaires, n^b^ (%)		
	Preconsultation PRO questionnaires sent to participants	162 (100)
	Preconsultation PRO questionnaires completed	94 (58)
Message platform, median (min-max; n)		
	Message sent from participants to clinic	1 (0-57; 592)
	Message sent from clinic to participants	1 (0-37; 532)
Individually tailored information section		
	Information section in use, n (%)	58 (32.6)
Reconsent		
	Reconsenting to app use, n (%)	95 (53.3)

^a^PRO: patient-reported outcome.

^b^The total number of questionnaires sent to the entire group.

### Self-Management

For the primary outcome (self-management measured with PAM-13), scores remained high from baseline to follow-up, with no observed statistically significant between-group difference in estimated mean change between the DigiDiaS care group and the usual care group after 1-year (MD 1.18, 95% CI –5.2 to 7.6; *P*=.72), estimated using a GLM (Tables 3 and 4). In total, 26 (21.31%) participants in the DigiDiaS care group and 10 (28.6%) in the usual care group increased by one or more levels of self-management during the 1-year follow-up. However, the proportions of participants who increased by one or more levels in both groups were not statistically significantly different (*P*=.37).

**Table 3:** Results from the generalized linear model analyses for continuous primary and secondary outcomes comparing DigiDiaS care and usual care (initial group choice analysis).

		DigiDiaS care			Usual care			Between groups			*P* value
		Participants, n	Estimated mean (95% CI)	Participants, n		Estimated mean (95% CI)	Participants, n		MD^a^ (95% CI)		
Self-management score (PAM-13)^b^											
	Baseline	164	71.1 (68.8 to 73.3)	47		71.1 (66.8 to 75.3)	—^c^		—	—	
	Follow-up	131	71.2 (68.6 to 73.7)	37		69.0 (64.1 to 73.8)	157		0.87 (–4.1 to 8.6)	.48	
HbA_1c_^d^											
	Baseline	184	59.9 (57.8 to 62.0)	51		54.3 (50.3 to 58.4)	—		—	—	
	Follow-up	134	57.8 (55.5 to 60.2)	51		53.4 (48.7 to 58.0)	—		–1.12 (–5.7 to 3.5)	.63	
Time in range											
	Baseline	152	62.6 (59.7 to 65.6)	41		64.5 (58.7 to 70.4)	—		—	—	
	Follow-up	126	62.8 (59.5 to 66.1)	30		62.0 (55.5 to 68.6)	—		2.65 (–4.7 to 10.0)	.48	
Well-being score (WHO-5)^e^											
	Baseline	164	60.3 (59.0 to 61.7)	47		61.2 (58.6 to 63.7)	—		—	—	
	Follow-up	132	61.0 (59.4 to 62.5)	36		59.4 (56.3 to 62.4)	—		2.47 (–1.8 to 6.8)	.26	

^a^MD: estimated between-group mean difference.

^b^PAM-13: Patient Activation Measure short version. Self-management (PAM-13): this scale ranges from 0 to 100, where higher scores indicate greater activation. Change is reported as the change in total score, mean (SD).

^c^Not available.

^d^HbA_1c_: glycated hemoglobin.

^e^WHO-5: Five Well-Being Index. This scale ranges from 0 to 100, where higher scores indicate better well-being. Scores below 50 indicate mild to severe depressive symptoms. Change is reported as the change in total score, mean (SD).

**Table 4:** Changes in categorical primary and secondary outcomes in DigiDiaS care and usual care during the 1-year follow-up (initial group choice analysis).

				DigiDiaS care, n/N (%)		Usual care, n/N (%)
Self-management levels (PAM-13)^a^						
	Level 1					
		Baseline	11/164 (5.9)		3/47 (5.8)	
		Follow-up	5/131 (2.7)		3/37 (5.8)	
	Level 2					
		Baseline	8/164 (4.3)		5/47 (9.6)	
		Follow-up	10/131 (5.4)		1/37 (1.9)	
	Level 3					
		Baseline	59/164 (31.9)		9/47 (17.3)	
		Follow-up	26/131 (14.1)		9/37 (17.2)	
	Level 4					
		Baseline	86/164 (46.5)		30/47 (57.7)	
		Follow-up	90/131 (48.6)		24/37 (46.2)	
		Change in level^b^	26/122 (21.3)		10/35 (28.6)	
HbA_1c_ above 75 mmol/mol^c^						
	Baseline		32/184 (17.3)		4/51 (7.7)	
	Follow-up		17/134 (9.2)		0/31 (0)	
	Change in level		12/134 (8.9)		3/31 (10)	
Well-being (WHO-5) score <50^d^						
	Baseline		40/164 (21.6)		6/47 (11.5)	
	Follow-up		39/132 (21.1)		5/36 (9.6)	
	Change in level		13/123 (10.5)		3/34 (8.8)	

^a^PAM-13: Patient Activation Measure short version. Self-management levels (PAM levels): the levels are categorized as follows: Level 1 is ≤47.0, Level 2 is 47.1-55.1, Level 3 is 55.2-67.0, and Level 4 is ≥67.1. A higher level indicates higher self-management. Change is reported as the number (%) of participants who moved up one or more levels from baseline to follow-up.

^b^*P*=.37.

^c^HbA_1c_: glycated hemoglobin. The treatment goal for HbA_1c_ in Norwegian people with type 1 diabetes is 53 mmol/mol, and values ≥75 mmol/mol are considered poor glycemic control. A change in HbA_1c_ of 5.5 mmol/mol is considered clinically relevant.

^d^WHO-5: Five Well-Being Index. Score <50: refers to the number of participants with scores below 50, indicating depressive symptoms. Change is reported as the number (%) of participants who improved from a score below 50 at baseline to a score above 50 at follow-up.

### Glycemic Control and Well-Being

For the secondary outcomes, glycemic control measured with HbA_1c_ (MD –2.1, 95% CI –6.6 to 2.4; *P*=.36), time in range (MD 5.77, 95% CI –1.53 to 13.1; *P*=.12) and well-being (MD 1.9, 95% CI –3.1 to 6.9; *P*=.46), no statistically significant between-group differences were observed between DigiDiaS care and usual care (Tables 3 and 4).

For details on other clinical and self-reported outcomes, see Multimedia Appendix 5 for categorical variables and unadjusted continuous outcomes and Multimedia Appendix 13 for adjusted continuous outcomes.

### Health Care Utilization

Health care utilization was examined in the DigiDiaS care and usual care groups, including attendance at individual consultations, group education, appointment changes, and nonattendance at scheduled consultations (Table 5). There were no statistically significant between-group differences in overall health care utilization during the 1-year follow-up. Specifically, 14 (7.9%) participants in the DigiDiaS care group and 8 (16.3%) in the usual care group did not attend any consultations or education courses during the follow-up period (*P*=.08).

There were no statistically significant between-group differences in consultations conducted with a diabetes specialist nurse (*P*=.15) or endocrinologist (*P*=.96). Video consultations were conducted exclusively in the DigiDiaS care group (n=14), whereas no statistically significant between-group differences were observed for in-person (*P*=.43) or telephone mode of consultations (*P*=.98). The rate of appointment changes did not differ statistically significantly between the DigiDiaS care and usual care group *(P=*.47). Further details are provided in Table 5.

The PRO-based questionnaire was distributed in 162 out of a total of 260 individual consultations with a diabetes specialist nurse or endocrinologist, corresponding to 62.3% of all consultations (Tables 2 and 5).

A total of 2 (1%) participants in the DigiDiaS care group were hospitalized once for ketoacidosis during the study period, each for 4 days. Additionally, 1 (0.5%) participant in the usual care group was hospitalized twice for ketoacidosis, first for 4 days and then for 21 days. No participants were hospitalized for hypoglycemia.

**Table 5:** Health care utilization in the DigiDiaS care and usual care groups during the 1-year follow-up.

		DigiDiaS care (n=178)	Usual care (n=50)	*P* value
Overall health care utilization, n (%)				
	Individual consultation attendance last year	159 (89.3)	42 (84)	.30
	Group education participation last year	51 (28.7)	8 (16.3)	.82
	No consultation or course engagement	14 (7.9)	8 (16.3)	.08
Individual consultation, n (%)		280 (100)	68 (100)	.30
	Diabetes specialist nurse	189 (67.5)	46 (67.6)	.15
	Endocrinologist	71 (25.3)	21 (30.8)	.96
	Clinical nutritionist	13 (4.6)	1 (1.4)	N/A^a^
	Consultations triggered by messages from participant	7 (2.5)	N/A	N/A
Mode of individual consultation, n (%)				
	In person	209 (74.6)	53 (77.9)	.43
	Telephone	57 (20.2)	15 (22.1)	.98
	Video	14 (5.0)	0 (0)	N/A
Group education, n (%)		61 (100)	11 (100)	.82
	Diabetes education course	5 (8.2)	2 (18.1)	N/A
	Carbohydrate counting	26 (42.6)	7 (63.6)	.91
	Pump	30 (49.2)	2 (18.1)	N/A
Change of appointment, n (%)		91 (100)	18 (100)	.47
	Patient change/cancellation of individual consultation or group education	64 (70.3)	13 (72.2)	.20
	The clinic has changed an appointment	27 (29.7)	5 (27.8)	N/A
Nonattendance, n (%)		16 (100)	3 (100)	N/A
	Nonattendance individual consultation	12 (75.0)	2 (66.6)	N/A
	Nonattendances at group education	4 (25.0)	1 (33.3)	N/A

^a^N/A: statistical analysis not applicable due to small numbers.

### Sensitivity Analysis

In the analysis according to initial group choice, the GLM model with primary and secondary outcomes adjusted for age, sex, and baseline HbA_1c_ (Multimedia Appendix 4) remained nonsignificant. In extreme-case sensitivity analyses of the primary outcome for initial group choice, the worst‑case scenario, the between‑group difference remained nonsignificant (MD 1.3, 95% CI –9.3 to 11.8; *P*=.81), and the best‑case scenario yielded a similar result (MD 1.3, 95% CI –5.5 to 8.3; *P*=.70). Results were comparable in the as-treated analysis, full details provided on extreme-case sensitivity analysis are provided in Multimedia Appendix 6.

In the as-treated sensitivity analysis, there were no statistically significant between-group differences in change over 1 year for the adjusted analysis on the primary outcome of self-management (*P*=.59) and secondary outcomes of glycemic control (HbA_1c_: *P*=.08; time in range: *P*=.09) and well-being (*P*=.85; Multimedia Appendices 7-9). For health care utilization in the as-treated allocation, analysis, and interpretation were limited by the small number of participants in the usual care group (n=32), and only a few variables were comparable (Multimedia Appendix 10). Under this sensitivity analysis, a higher proportion of participants in the DigiDiaS care group (n=176, 89.8%) had one or more individual consultations compared with usual care (n=25, 78.1%; *P*=.04). In addition, the DigiDiaS care group had statistically significantly more individual consultations (DigiDiaS care: n=310; usual care: n=32; *P*=.04) and more nurse-led consultations (DigiDiaS care: n=212; usual care: n=23; *P*=.006). These as‑treated findings are exploratory and should be interpreted with caution, given self‑selection, group switching, and small cell counts in usual care.

## Discussion

### Principal Findings

Under routine conditions, this longitudinal real-world pragmatic observational study compared DigiDiaS care, a flexible digital mobile health supplement to type 1 diabetes outpatient care, with usual care over 1-year. The primary outcome was self-management, with secondary outcomes including glycemic control, well-being, health care utilization, and use of the DigiDiaS platform. Given the self-selection into DigiDiaS care or usual care and group switching during follow-up, the between-group comparisons are presented as exploratory. Based on participants’ initial group choice, no statistically significant between-group differences between DigiDiaS care and usual care in self-management, glycemic control, or well-being over 1-year were detected. The as-treated analyses supported these findings. For health care utilization, the primary analysis according to initial group choice showed no statistically significant between-group differences. However, in the as-treated sensitivity analysis, the number of individual consultations was statistically significantly higher in the DigiDiaS care group than in the usual care group. These utilization findings are exploratory and hypothesis-generating and should be interpreted cautiously, given the small numbers in usual care. As expected, participants in the DigiDiaS care group interacted with the digital solution in different ways. Although no statistically significant between-group differences were detected, the findings raise several interesting points to discuss.

### Comparison With Previous Evidence

In our study, there were no between-group differences in changes in primary, secondary, or other health outcomes over 1-year compared with usual care. Overall, this aligns with previous research showing that digital health interventions often achieve outcomes comparable to traditional care for chronic conditions, including type 1 diabetes [54]. The choice of self-management (PAM‑13) as the primary outcome was prespecified in the protocol, reflecting the intended mechanism of DigiDiaS care, which is to reorganize outpatient care to detect needs in a timely manner and target support through more focused consultations, with low‑threshold access. In practice, several factors may have limited sensitivity to change in self-management over 1-year. We found that the self-management activation levels were already high at baseline; thus, the early evaluation of the implementation of digital outpatient care in development may not have provided sufficient “dose” to shift a global self‑management index. Similarly, the selection into the study may have favored participants with greater capacity and higher baseline self‑management. Taken together, neutral findings on self-management are therefore plausible. For the clinic, it was particularly important that the introduction of digital outpatient care did not deteriorate glycemic control. The lack of clear deterioration in HbA_1c_ is therefore reassuring, although the estimates do not exclude clinically relevant changes. Digital platforms such as DigiDiaS may have the potential to maintain care quality while offering more flexible, patient-centered approaches [55]. In Norwegian outpatient diabetes care, increasing pressure on the health care system and sustainability considerations make it important to maintain quality while exploring innovative digital solutions that support flexible, patient-centered care [56].

At baseline inclusion, all participants were introduced to the new digital outpatient care model, and the real-world study design allowed them to choose DigiDiaS care or usual care and to switch groups later if desired. During the 1-year follow-up in our study, 18 participants opted to transition from usual care to DigiDiaS care. Transitions between groups may reflect adaptation dynamics (eg, that some patients need more time to adopt a new digital outpatient care model) as well as baseline imbalance between DigiDiaS care and usual care. At baseline, participants in the usual care group had statistically significantly longer diabetes duration, higher well-being, and a lower proportion of insulin pump users than those in the DigiDiaS care group [21]. Participants in the usual care group, who were less familiar with diabetes-related technology, may therefore have needed more time to adapt and recognize the possible benefits of DigiDiaS care. During insulin pump group education, health care professionals encouraged patients to join DigiDiaS care as they regarded it as a positive supplement specifically for those using insulin pumps, and the as-treated analysis showed that all usual care participants attending such insulin pump group education transitioned to DigiDiaS care. As previously noted, insights from the qualitative study of DigiDiaS care indicate that health care professionals’ introduction and acceptance of the solution influenced patients’ willingness to adopt the new care model [22]. Consistent with previous research, health care professionals’ recommendations and patients’ recognition of a digital solution’s value are key factors in adoption [22,57]. Participants switching groups highlight the importance of understanding how DigiDiaS care and similar digital health solutions are used in clinical practice by both patients and health care professionals, including the extent of engagement with the platform and how its features are introduced and utilized.

### Utilization of the Digital Platform

The premise for using the DigiDiaS care platform was each participant’s need, whether self-identified or recognized by the health care service. Over 80% (n=145, 81%) of participants used the DigiDiaS care platform during the study period. As anticipated, use varied substantially, ranging from those who logged in once, completed a single PRO-based questionnaire, or sent 1 message, to participants who used multiple features (PRO-based questionnaires, asynchronous messaging, video consultations, and the individually tailored information section in the app) more extensively. Reconsent was counted as an access event and as app use because it reflects a confirmed intentional viewing of the individually tailored information page or access to sent or received messages. Given that DigiDiaS care is driven by clinical support needs and scheduled consultations, substantial variation in use was expected [20]. Not all participants were scheduled for a consultation during the 1-year follow-up and, therefore, did not receive the preconsultation PRO-based questionnaires. Among those who did receive a PRO-based questionnaire, 58% (94/162) responded. If participants did not respond to the PRO-based questionnaire, 2 automatic reminders were sent. Our qualitative study highlights that both patients and health care professionals recognized that the PRO-based questionnaires can be a valuable tool for setting the agenda and addressing patients’ needs during consultations [22]. However, we observed inconsistent use of the PRO-based questionnaires in this study. Some health care professionals did not consistently introduce or explain their purpose, particularly at first use, and did not always refer to them during consultations, which may have reduced their perceived relevance and importance [22]. The inconsistent use and variation in PRO-based questionnaire distribution and response should be interpreted in the context of this real-world implementation of DigiDiaS care. Although the system had been in routine use for a year before recruitment to this study began, full adoption and embedding into the clinic workflow takes time [58,59]. In practice, PRO-based questionnaire distribution was manual and triggered when appointments were scheduled. Lack of automation, workflows that were still maturing, and human error meant that PRO‑based questionnaires were distributed for only 162 of 260 individual consultations with a diabetes specialist nurse or an endocrinologist in the DigiDiaS care group. Ensuring consistent distribution of PRO-based questionnaires and clinician engagement with responses, alongside reminders, may be crucial to support meaningful use of PRO-based questionnaires and encourage patient participation [22,60]. In a needs‑triggered model such as DigiDiaS care, universal completion is not the aim; however, consistent distribution is important for distinguishing lack of need from nonengagement.

Among the platform’s features, the asynchronous messaging feature was most utilized, with about half of the participants contacting the health care professionals during the study period, and a subset used the feature extensively. Previous studies similarly reported substantial variety, suggesting that patient needs and individual preferences influence usage patterns [61-63]. Asynchronous messaging allows for closer contact with the health service outside planned consultations and was especially highlighted in our qualitative work as facilitating individualized care [22]. Coping with type 1 diabetes requires substantial self-management, and several studies suggest that digital contact with health care services can support a sense of security [22,64,65]. Sense of security and digital contact with health care services may be particularly valuable during changes in insulin regimens or the initiation of insulin pumps, when support can help patients navigate challenges and reduce concerns and burden [54,66]. Norwegian diabetes specialist nurses have also emphasized the importance of easy access to support and communication with health care providers during insulin pump initiation [67]. None of our measured outcomes captured patients’ sense of security associated with access to DigiDiaS care. This sense of security may involve reassurance of mutual engagement between patients and health care professionals, and confidence that support is available when needed [68]. We may therefore have overlooked an important difference between DigiDiaS care and usual care. Measuring sense of security in digital health is challenging because validated instruments for this context are lacking, and existing literature is predominantly qualitative [69]. In our qualitative work, patients described asynchronous messaging as lowering the threshold for contact and providing reassurance that help was “just a message away” [22]. Messaging may also help to free health care resources by addressing questions outside scheduled consultations; however, its impact on utilization remains unclear [70] and should be investigated further. Taken together, our observations and previous research highlight how messaging may complement traditional care models by providing timely support during critical periods and tailoring contact to individual patient needs [8,13].

### Limitations and Strengths

This study has several limitations and strengths. The nonrandomized design limits between-group comparability due to potential confounding. Allowing participants to choose their care model and switch groups during the study period reflects real-world practice, in which patients and health care services adapt to novel digital outpatient care models. This flexibility provided valuable insight into how the digital solution was utilized in routine care without researcher interference and captured how patients made choices based on their needs. However, modest between-group differences may have been present but undetected due to limited statistical power. In addition, the uneven distribution of participants between groups created challenges in interpreting between-group changes from baseline to follow-up. To address these limitations and check for possible confounding, we fitted GLM models, unadjusted and adjusted for prespecified baseline covariates (diabetes duration, insulin delivery method, and WHO‑5 well‑being). The similarity of adjusted and unadjusted estimates supports the robustness of our findings. Due to limited statistical power, we prioritized adjusting the model for known baseline differences and added a second GLM with other relevant covariates in Multimedia Appendix 4. We also conducted both initial group choice and as-treated analyses to account for group switching, enabling a more nuanced interpretation of the findings. Another limitation was the unidirectional approach used to analyze categorical variables, which focused exclusively on predefined positive changes or desired outcomes. Although this approach simplified the analysis and emphasized clinically meaningful improvements, it did not capture changes in the opposite direction (eg, declines in self-management levels) or nuanced fluctuations over time. In addition, digital health literacy was measured only at follow-up, and change over time could not be assessed. Furthermore, incomplete distribution of PRO-based questionnaires may have reduced the implementation fidelity of DigiDiaS care. Missing data from electronic health records and self-reported questionnaires posed challenges, as distribution of follow-up questionnaires and data extraction did not always align with outpatient clinic appointments, which affected the availability of measurements and blood tests. Allowing participants to complete questionnaires either digitally or on paper accommodated individual preferences and may have increased participation [60]. However, the paper format allowed skipped items and, therefore, increased missingness. To address this, we reported the number of responses for each variable. The proportion of nonresponders to the self-reported questionnaire was 29% (52/178) in the DigiDiaS care group, which was approximately twice that in the usual care group (7/50, 14%) and likely reflects the larger share of participants in the DigiDiaS care group. To address challenges related to nonresponders, we also conducted an extreme-case sensitivity analysis on the primary outcome that did not change the results. The response rate in the DigiDiaS care group for a questionnaire administered both digitally and on paper was consistent with published rates. Despite these challenges, the study design aimed to accommodate individual preferences and achieve an adequate response rate by offering completion options and using measures such as reminders to complete the questionnaires and the flexibility to choose between digital and paper formats. The digital format supported completeness through mandatory responses and easy access, whereas the paper format increased inclusiveness for those participants less comfortable with digital tools.

### Conclusion

In this real-world pragmatic observational comparison conducted under routine conditions, outpatient care for patients with type 1 diabetes was reorganized into a flexible digital model, DigiDiaS care. Compared with usual care, no statistically significant between-group differences in changes were observed for the primary outcome of self-management or the secondary outcomes of glycemic control and well-being. We also found no statistically significant differences in health care utilization between DigiDiaS care and usual care. Over 80% (n=145, 81.5%) of participants who opted for DigiDiaS care engaged with the digital platform, with patient-initiated asynchronous messaging being the most frequently used feature. Given the self-selection of care model and that some patients changed the model of care they received during the study, the results should be interpreted as exploratory. The findings indicate that a flexible digital outpatient care model may maintain key outcomes while increasing flexibility for both patients and providers. However, the successful adoption and implementation of digital outpatient care models in real-world settings requires attention to the diverse needs and experiences of users to maximize potential impact.

## Appendix

Multimedia Appendix 1 Deviations from the study protocol.

Multimedia Appendix 2 Screenshot from patient-reported outcome–based questionnaire and asynchronous message feature.

Multimedia Appendix 3 Details on measures: demographic and clinical variables, and self-management, well-being, diabetes distress, health literacy, experience of involvement, and digital health literacy.

Multimedia Appendix 4 Initial group choice: generalized linear model adjusted for age, gender, and baseline glycated hemoglobin.

Multimedia Appendix 5 Initial group choice: unadjusted generalized linear model, change from baseline to follow-up primary and secondary outcomes.

Multimedia Appendix 6 Initial group choice: extreme-case sensitivity analysis in primary outcome.

Multimedia Appendix 7 As-treated: use of features in DigiDiaS care.

Multimedia Appendix 8 As-treated: boxplot for messages sent from patients.

Multimedia Appendix 9 As-treated: unadjusted analysis: change from baseline to follow-up primary and secondary outcomes.

Multimedia Appendix 10 As-treated: health care use.

Multimedia Appendix 11 Initial group choice: responders and nonresponders.

Multimedia Appendix 12 Initial group choice and as-treated: digital health literacy: Health Literacy Survey 2019-2021, digital skills, Norwegian part.

Multimedia Appendix 13 Initial group choice: generalized linear model adjusted for covariates for other clinical and self-reported outcomes.

## Abbreviations

GLM: generalized linear model
HbA1c: glycated hemoglobin
HLS19-DHC-NO_Norwegian: Health Literacy Survey 2019-2021, digital skills, Norwegian part
HLS19-Q12: short version of the European Health Literacy Survey Questionnaire
MD: between-group mean difference
NOKLUS: The Norwegian Organization for Quality Improvement of Laboratory Examinations
PAID-20: Problem Areas in Diabetes
PAM-13: Patient Activation Measure short version
PRO: patient-reported outcome
PROM: patient-reported outcome measure
STROBE: Strengthening the Reporting of Observational Studies in Epidemiology
TSD: services for sensitive data
WHO-5: Five Well-Being Index

## Data Availability

The datasets generated or analyzed during this study are available from the corresponding author on reasonable request.
