# Supplementary material for: Digital Outpatient Care for Patients With Type 1 Diabetes (DigiDiaS): Pragmatic Observational Pre-Post Study
Source: J Med Internet Res. 2026 Jul 13;28:e94782. doi: 10.2196/94782 (PMC13408466; doi:10.2196/94782)
Supplement: Multimedia Appendix 1 [file jmir_v28i1e94782_app1.docx]

### Supplement 1: Deviations from the study protocol

Supplement 1: Deviation from the study protocol and what was carried out in the current study.

| Study protocol | Current study |
| --- | --- |
| Reporting of the proposed study and participation in digital tools and PROMs in clinical practice will be guided by better reporting of interventions: template for intervention description and replication (TIDieR) and the PRO reporting guide. | Reporting was guided by the STrengthening the Reporting of OBservational studies in Epidemiology (STROBE) guideline. |
| The group that does not receive DigiDias care is described as "standard care". | The group that does not receive DigiDiaS care was described as “Usual care”. |
| A second researcher world control 10 % of data extractions. | A second researcher controlled 100% of the data extractions. |
| Tobacco use would be extracted at baseline and follow-up. | Tobacco use was extracted at baseline only. |
| Late complications from diabetes (albuminuria, treated with dialysis, transplanted kidney, retinopathy, neuropathy, stroke, arterial vascular surgery, amputation, and diabetic foot ulcers) would be extracted at baseline only. | Late complications from diabetes were extracted at baseline and follow-up. |
| Diabetic ketoacidosis, hypoglycemia in need of help and symptomatic hypoglycemia would be extracted from medical records at baseline and follow-up. | Ketoacidosis, hypoglycemia in need of help and symptomatic hypoglycemia were extracted from medical records at baseline. At follow-up, hospital admissions for ketoacidosis and hypoglycaemia during the follow-up period were extracted from the electronic health records system using diagnosis code. |
| Frequency of and number of messages sent via the asynchronous message service would be extracted. | Number of messages from patients to healthcare professionals and from healthcare professionals to patients were extracted. Frequency of use was not extracted. |
| Differences in mean changes in short- and long-term variables would be modeled using ANOVA. Logistic regression would adjust for age, gender, and education as possible confounders. | Differences in change were calculated using a generalised linear model (GLM) with repeated‑measures linear mixed‑effects, reported as estimated mean between-group difference (MD), with 95% CI and P-values. The primary analysis was adjusted for baseline differences, diabetes duration, insulin delivery method, and WHO-5 well-being score. A secondary analysis were adjusted for HbA1c, gender and age. |
