# Supplementary material for: Digital Outpatient Care for Patients With Type 1 Diabetes (DigiDiaS): Pragmatic Observational Pre-Post Study
Source: J Med Internet Res. 2026 Jul 13;28:e94782. doi: 10.2196/94782 (PMC13408466; doi:10.2196/94782)
Supplement: Multimedia Appendix 2 [file jmir_v28i1e94782_app2.docx]

### Supplement 2: Screen shot from PRO-based questionnaire and asynchronous message feature

Previously published by Mollestad et al., 2025 [22].


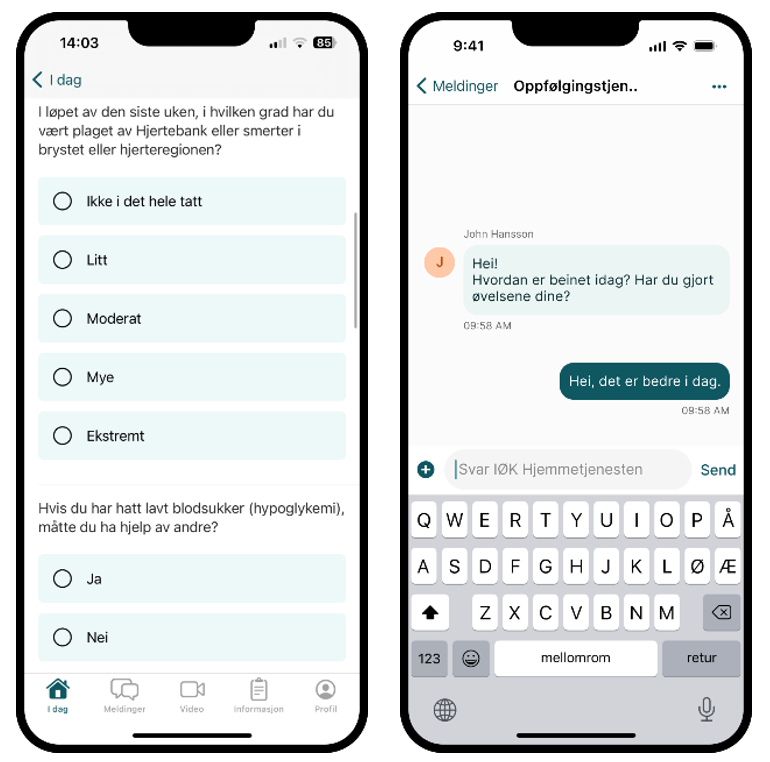


The left screenshot illustrates sample items from the pre-consultation patient-reported outcome questionnaire in the app. The right screenshot presents an example chat conversation in the asynchronous messaging feature. The images are taken from a demonstration account and are for illustrative purposes. No real patient data are displayed.
