# Supplementary material for: Digital Outpatient Care for Patients With Type 1 Diabetes (DigiDiaS): Pragmatic Observational Pre-Post Study
Source: J Med Internet Res. 2026 Jul 13;28:e94782. doi: 10.2196/94782 (PMC13408466; doi:10.2196/94782)
Supplement: Multimedia Appendix 3 [file jmir_v28i1e94782_app3.docx]

### Supplement 3: Detail on measures: demographic and clinical variables, and self-management, well-being, diabetes distress, health literacy, experience of involvement and digital health literacy

Supplement 3: Details on measures, demographic and clinical variables (Supplement 3A), and self-management, well-being, diabetes distress, health literacy, experience of involvement and digital health literacy (Supplement 3B).

| Supplement 3A: Details on measures, demographic and clinical variables | | | | |
| --- | --- | --- | --- | --- |
| **Variable** | **Categories or data type** | **Merged into categories for analysis** | **Interpretation and estimands** | **Data collection point** |
| Gender | Woman or man. | N/A | N/A | Baseline |
| Age | Years. | N/A | N/A | Baseline |
| Education | Not completed primary school (10 years), Primary upper secondary school (13 years), Vocational school (13 years), College or university (≤ 4 years), College or university (> 4 years), unknown. | Recoded into Education up to upper secondary school or vocational school (13 years), University/college education up to 4 years, University/college education more than 4 years. | N/A | Baseline |
| Diabetes duration | Time from diabetes onset to the date the participant provided study consent, in years. | N/A | N/A | Baseline |
| HbA_1c_ | mmol/mol on a continuous scale. | N/A | The treatment target is 53 mmol/mol, and values ≥ 75 mmol/mol are considered poor glycaemic control [1]. Change is reported as mean (SD). Higher HbA1c indicates poorer glycaemic control, whereas lower HbA1c indicates improved control. | Baseline and follow-up |
| HbA_1c_  ≥ 75 mmol/mol | HbA_1c_ Above or below 75 mmol/mol. | N/A | Count and percentage at baseline.  Change is reported as the count and percentage of participants that had HbA_1c_ above 75mmol/mol at baseline but at 1-year follow-up is now below. | Baseline and follow-up |
| Insulin delivery | Pump or pen. | N/A | Count and percentage at baseline.  Change is reported as the count and percentage of participants that had insulin pen at baseline and after 1-year follow-up have an insulin pump. | Baseline and follow-up |
| Blood glucose monitoring | Continuous glucose monitoring (CGM) or glucometer. | N/A | Count and percentage at baseline.  Change is reported as Count and percentage that used glucometer at baseline and after 1-year follow-up use a CGM. | Baseline and follow-up |
| Time in range, for those using CGM | Percentage of Time in Range (3.9-10 mmol/mol) last 14 days. | N/A | Change is reported as mean (SD). Positive scores indicate a longer proportion of time within the range, while negative scores indicate a shorter proportion of time within the range. | Baseline and follow-up |
| Hypoglycaemia | Never, once, several times, or unknown. | Recoded into none, one or more times, missing | Count and percentage at baseline. | Baseline |
| Diabetic ketoacidosis | Never, once, Several times, or unknown. | Recoded into none, one or more times, missing | Count and percentage at baseline. | Baseline |
| Late complications from diabetes | Albuminuria, Retinopathy, Neuropathy, Diabetic foot ulcers, Stroke, Arterial vascular surgery, Dialysis treatment, Kidney transplantation, Amputation. | Recoded into none, one, two, or more, and missing. | Count and percentage. | Baseline and follow-up |
| Comorbidities | Charlson Comorbidity Index (CCI) [2], which includes a range of conditions such as myocardial infarction, congestive heart failure, peripheral vascular disease, and others. | None, one, two, or more, or missing. | Count and percentage at baseline. | Baseline |
| Blood pressure | Systolic blood pressure and  Diastolic blood pressure in mmHg. | N/A | Treatment goal for persons with type 1 diabetes for blood pressure is 135/85 mmHg [1].  Change is reported as Mean (SD). Positive score for increased systolic/diastolic blood pressure and a negative score for decreased. | Baseline and follow-up |
| Cholesterol | Low-Density Lipoprotein (LDL). | N/A | LDL over 2.5 mmol/mol indicates the need for medication for patients aged 40 - 80 years with no known cardiovascular disease, and for patients < 40 years with other risk factors for persons with type 1 diabetes [3].  Change is reported as mean (SD). Positive values indicate increased LDL cholesterol, and negative values indicate decreased LDL cholesterol. | Baseline and follow-up |
| BMI | Weight and height. | BMI=weight (kg) / [height (m)]^2. | N/A | Baseline |

| Supplement 3B: Details on self-management, well-being, diabetes distress, health literacy, experience of involvement and digital health literacy | | | | |
| --- | --- | --- | --- | --- |
| **Phenomena**  **and measure** | **Items, scale, domains** | **Interpretation** | **Estimands^a^** | **Data collection point** |
| Self-management  (PAM-13) | 13 items, rated from 1 'disagree strongly,' to 4, 'strongly agree,' with an additional 'not applicable'.  Four domains: knowledge, beliefs, confidence, and skills for managing one's health. | The total PAM-13 score is calculated using a formula that weighs the various questions and that forms a scale from 0 to 100. Higher scores indicate higher activation [4].  Four levels and scores [5]:  Level 1 (≤47.0): disengaged and overwhelmed  Level 2 (47.1-55.1): becoming aware but still struggling  Level 3 (55.2–67.0): taking action  Level 4 (≥67.1): maintaining behaviour and pushing further.  Levels 1 and 2, 3 and 4 were merged into categories for analysis. | For the categorical levels, number (%) of participants that went from one level up to the next or higher from baseline to follow-up is reported.  A 3.2 change on a 1-100 scale is considered a small clinically relevant change [6] | Baseline and follow-up |
| Well-being  (WHO-5) | 5 items, measured on a 6-point Likert scale from 0 ‘never’ to 5 ‘all the time’. | Sum of 5 items multiplied by 4 to create a scale from 0-100 [7].  A higher score indicates higher well-being.  Score < 50 indicates a mild to severe depressive affects [8]. | For the categorical variable, number (%) of participants that went from score below 50 at baseline to score above 50 at follow-up is reported as count and percentage. | Baseline and follow-up |
| Diabetes distress  (PAID) | 20 items, ranked on a 5-point Likert scale from 0, ‘not a problem’ to 4, ‘serious problem’. | Sum of all 20 items and multiplied by 1.25, giving a total score ranging from 0 to 100 [9].  A higher score reflects greater emotional distress. A score of 40 or above indicates severe emotional distress. | For the categorical variable, number (%) of participants that went from a score above at baseline to a score below 40 at follow-up is reported. | Baseline and follow-up |
| Health literacy  (HLS19–Q12) | The 12 items are measured in a 4-point Likert scale from 1, ‘very hard’ to 4, ‘very easy’ with added ‘I don’t know’.  Four levels: Below level 1, level 1, level 2, and level 3.  Three domains:  healthcare (HC), disease prevention (DP), and health promotion (HP). | Sum score of the 12 items is calculated. Higher scores reflect higher health literacy [10].  The domain characteristics:  Below level 1 (<27): Lack key knowledge and skills about health and may have challenges in understanding and applying health information Level 1 (27-32): level 1 where you are expected to be able to access, understand and apply health information relevant to staying healthy.  Level 2 (33-38): access, appraise, understand, and apply health information and advice relevant to enhancing physical and mental health.  Level 3 (≥39): access, appraise, understand, and apply health information and advice relevant to making informed healthcare choices by critically evaluating health claims and judiciously comparing treatments.  The items can also be distributed into the three domains, and the individual domain can be scored with a minimum of points 0 maximum of 16 points per domain. | For the categorical levels, the number (%) of participants that went from one level up to the next or higher from baseline to follow-up is reported. | Baseline and follow-up |
| Digital health literacy | The 10- items are rated on a 4-point Likert scale ranging from 1 (‘very hard’) to 4 (‘very easy’) with an additional ‘I don’t know’ option. | We compared proportions with the highest and lowest scores on each item between the groups [11]. | N/A | Follow-up |
| Experience of involvement | The 5 items measure participants’ level of agreement with the statements on a 6-point scale, ranging from 0 (*not at all*) to 5 (a very high degree).  1. The healthcare professionals asked questions about my experiences with the disease. 2. I talked to healthcare professionals about the questions and concerns that I had. 3. The healthcare professionals invited me to ask questions and talk about my concerns. 4. I was consulted when decisions about my plans were made. 5. I talked adequately to healthcare professionals about how I manage my condition. | The items were summed to a score ranging from 0 (worst) to 25 (best) [12]. |  | Baseline and follow-up |

| a - Change in continuous outcomes is reported as estimated means with 95% confidence intervals at baseline and at follow‑up, and as the between‑group mean difference (MD) in change with 95% confidence intervals. |
| --- |

**References to supplement 3**

1. Norwegian Directorate of Health. Treatment targets for type 1 diabetes. Oslo: Norwegian Directorate of Health; 2016. Updated December 5, 2024. Accessed February 17, 2025. URL: https://www.helsedirektoratet.no/retningslinjer/diabetes/behandling-med-blodsukkersenkende-legemidler-ved-diabetes/insulinbehandling-og-behandlingsmal-ved-diabetes-type-1/behandlingsmal-ved-diabetes-type-1

2. Charlson ME, Pompei P, Ales KL, MacKenzie CR. A new method of classifying prognostic comorbidity in longitudinal studies: development and validation. J Chronic Dis. 1987;40(5):373-83. PMID: 3558716. doi: 10.1016/0021-9681(87)90171-8.

3. Norwegian Directorate of Health. National professional guideline for diabetes [Internet]. Oslo: Norwegian Directorate of Health; 2016 Sep 14. Last updated June 2, 2025. Accessed January 6, 2026. URL: https://www.helsedirektoratet.no/retningslinjer/diabetes

4. Hibbard JH, Stockard J, Mahoney ER, Tusler M. Development of the Patient Activation Measure (PAM): conceptualizing and measuring activation in patients and consumers. Health Serv Res. 2004;39(4 Pt 1):1005-26. PMID: 15230939. doi: 10.1111/j.1475-6773.2004.00269.x.
5. Moljord IE, Lara-Cabrera ML, Perestelo-Pérez L, Rivero-Santana A, Eriksen L, Linaker OM. Psychometric properties of the Patient Activation Measure-13 among out-patients waiting for mental health treatment: A validation study in Norway. Patient Educ Couns. 2015;98(11):1410-7. PMID: 26146239. doi: 10.1016/j.pec.2015.06.009.
6. Steinsbekk A. Måling av effekt av pasientopplæring. J Norwegian Med Assoc. Oct 23, 2008;128(20):2316-2324.
7. de Wit M, Pouwer F, Gemke RJBJ, Delemarre-van de Waal HA, Snoek FJ. Validation of the WHO-5 Well-Being Index in Adolescents With Type 1 Diabetes. Diabetes Care. 2007;30(8):2003-6. PMID: 17475940. doi: 10.2337/dc07-0447.
8. Newnham EA, Hooke GR, Page AC. Monitoring treatment response and outcomes using the World Health Organization's Wellbeing Index in psychiatric care. J Affect Disord. 2010;122(1-2):133-8. PMID: 19592116. doi: 10.1016/j.jad.2009.06.005.
9. Polonsky WH, Anderson BJ, Lohrer PA, Welch G, Jacobson AM, Aponte JE, et al. Assessment of Diabetes-Related Distress. Diabetes Care. 1995;18(6):754-60. PMID: 7555499. doi: 10.2337/diacare.18.6.754.
10. Guttersrud Ø, Le C, Pettersen KS, Helseth S, Finbråten HS. Towards a progression of health literacy skills: establishing the HLS-Q12 cutoff scores. Res Square. 2019:1-20.
11. Le C, Finbråten HS, Griebler R, Levin-Zamir D, Guttersrud Ø. Ability to Utilize Digital Health Services: Validation of the Digital HealthCare Scale in Adolescents and Young Adults. HLRP Health Lit Res Pract. 2025;9(1):e19-e28. PMID: 39933534. doi:10.3928/24748307-20241204-01.
12. DEFACTUM®. Selection of patient involvement questions. Denmark: DEFACTUM®; 2016. Updated November 24, 2020. URL: http://www.defactum.dk/
