# Supplementary material for: Digital Outpatient Care for Patients With Type 1 Diabetes (DigiDiaS): Pragmatic Observational Pre-Post Study
Source: J Med Internet Res. 2026 Jul 13;28:e94782. doi: 10.2196/94782 (PMC13408466; doi:10.2196/94782)
Supplement: Multimedia Appendix 4 [file jmir_v28i1e94782_app4.docx]

Supplement 4: Initial group choice: GLM adjusted for age, gender, and baseline HbA1c

Supplement 4: Initial group choice on between-group change from baseline to follow-up on continuous outcomes including primary and secondary outcomes, disease specific variables and self-reported outcomes adjusted for age, gender and baseline HbA1c.

|  |  | **DigiDiaS care** | | **Usual care** | | **Between groups** | |  |
| --- | --- | --- | --- | --- | --- | --- | --- | --- |
|  | | **N** | **Estimated mean**  **[95% CI]** | **N** | **Estimated mean**  **[95% CI]** | **N** | **MD [95% CI]** | ***P*** |
| **Self-management score (PAM-13)^a^** | | | | | | | | |
|  | Baseline | 164 | 71.0 [68.7 to 73.2] | 47 | 72.0 [67.7 to 76.3] |  |  |  |
|  | Follow-up | 131 | 71.1 [68.6 to 73.6] | 37 | 71.3 [66.5 to 76.1] | 157 | 0.8 [-5.6 to 7.2] | .807 |
| **HbA_1c_** | | | | | | | | |
|  | Baseline | 184 | 60.3 [59.3 to 61.4] | 51 | 58.9 [56.8 to 60.9] |  |  |  |
|  | Follow-up | 134 | 57.3 [56.1 to 58.5] | 51 | 57.9 [55.3 to 60.5] |  | -2.1 [-5.7 to 1.6] | .263 |
| **Time in range** | | | | | | | | |
|  | Baseline | 152 | 61.3 [58.8 to 63.9] | 41 | 60.7 [55.6 to 65.8] |  |  |  |
|  | Follow-up | 126 | 63.7 [60.9 to 66.5] | 30 | 57.5 [51.7 to 63.4] |  | 5.5 [-1.7 to 12.8] | .135 |
| **Well-being score (WHO-5)** | | | | | | | | |
|  | Baseline | 164 | 59.8 [57.1 to 62.4] | 47 | 63.1 [58.1 to 68.1] |  |  |  |
|  | Follow-up | 132 | 60.5 [57.7 to 63.2] | 36 | 62.3 [56.9 to 67.6] |  | 1.6 [-3.4 to 6.6] | .536 |
| **LDL-cholesterol** | | | | | | | | |
|  | Baseline | 183 | 2.6 [2.4 to 2.7] | 52 | 2.4 [2.2 to 2.6] |  |  |  |
|  | Follow-up | 98 | 2.4 [2.3 to 2.6] | 20 | 2.2 [1.8 to 2.5] |  | 0.1 [-0.3 to 0.5] | .667 |
| **Blood pressure systolic, mmHg** | | | | | | | | |
|  | Baseline | 172 | 132.6 [130.5 to 134.7] | 47 | 129.8 [125.5 to 134.1] |  |  |  |
|  | Follow-up | 74 | 133.7 [130.5 to 136.9] | 17 | 128.7 [121.8 to 135.5] |  | 2.3 [-5.9 to 10.4] | .586 |
| **Blood pressure diastolic, mmHg** | | | | | | | | |
|  | Baseline | 172 | 79.5 [78.3 to 80.7] | 47 | 77.5 [75.0 to 79.9] |  |  |  |
|  | Follow-up | 74 | 80.4 [78.6 to 82.1] | 17 | 76.9 [73.0 to 80.8] |  | 1.4 [-3.1 to 5.9] | .534 |
| **Diabetes distress score (PAID)** | | | | | | | | |
|  | Baseline | 164 | 25.4 [22.9 to 27.8] | 47 | 23.9 [19.2 to 28.7] |  |  |  |
|  | Follow-up | 129 | 24.3 [21.6 to 26.9] | 37 | 20.9 [15.8 to 25.9] |  | 2.0 [-2.8 to 6.7] | .424 |
| **Health literacy score (HLS19-Q12)** | | | | | | | | |
|  | Baseline | 164 | 33.9 [32.9 to 34.0] | 47 | 34.2 [32.3 to 36.2] |  |  |  |
|  | Follow-up | 132 | 34.8 [33.7 to 35.9] | 37 | 34.6 [32.5 to 36.7] |  | 0.5 [-1.8 to 2.8] | .655 |
