# Supplementary material for: Digital Outpatient Care for Patients With Type 1 Diabetes (DigiDiaS): Pragmatic Observational Pre-Post Study
Source: J Med Internet Res. 2026 Jul 13;28:e94782. doi: 10.2196/94782 (PMC13408466; doi:10.2196/94782)
Supplement: Multimedia Appendix 5 [file jmir_v28i1e94782_app5.docx]

### Supplement 5: Initial group choice: Unadjusted GLM, change from baseline to follow-up primary and secondary outcomes

Supplement 5: Initial group choice on between-group change from baseline to follow-up on primary and secondary outcomes (Supplement 5A), disease specific variables (Supplement 5B) and self-reported outcomes (Supplement 5C)

| Supplement 5A: Results from the GLM analyses for continuous and categorical outcomes between DigiDiaS care and usual care on the primary and secondary outcomes (initial group choice analysis). | | | | | | | | |
| --- | --- | --- | --- | --- | --- | --- | --- | --- |
|  |  | **DigiDiaS care** | | **Usual care** | | **Between groups** | |  |
| **Primary outcome** | | | | | | | | |
|  | | **N** | **Estimated mean**  **[95% CI]** | **N** | **Estimated mean**  **[95% CI]** | **N** | **MD^f^**  **[95% CI]** | ***P*** |
| **Self-management score (PAM-13)^a^** | | | | | | | | |
|  | Baseline | 164 | 70.8 [68.6 to 73.1] | 47 | 72.2 [67.9 to 76.4] |  |  |  |
|  | Follow-up | 131 | 71.3 [68.8 to 73.8] | 37 | 71.4 [66.7 to 76.1] | 157 | 1.18 [-5.2 to 7.6] | .718 |
|  |  |  | **n (%)** |  | **n (%)** |  |  |  |
| **Secondary outcomes** | | | | | | | | |
|  |  |  | **Estimated mean**  **[95% CI]** |  | **Estimated mean**  **[95% CI]** |  |  |  |
| **HbA_1c_^c^** | | | | | | | | |
|  | Baseline | 184 | 60.9 [58.9 to 62.9] | 51 | 55.2 [51.4 to 59.1] |  |  |  |
|  | Follow-up | 134 | 58.1 [55.9 to 60.3] | 31 | 54.5 [50.0 to 59.0] | 164 | -2.1 [-6.6 to 2.4] | .362 |
|  | |  | **n (%)** |  | **n (%)** |  |  |  |
|  |  |  | **Estimated mean**  **[95% CI]** |  | **Estimated mean**  **[95% CI]** |  |  |  |
| **Time in range** | | | | | | | | |
|  | Baseline | 152 | 60.6 [57.6 to 63.5] | 41 | 63.4 [57.7 to 69.2] |  |  |  |
|  | Follow-up | 126 | 63.1 [59.9 to 66.3] | 30 | 60.2 [53.7 to 66.6] | 134 | 5.77 [-1.5 to 13.1] | .121 |
| **Well-being score (WHO-5)^d^** | | | | | | | | |
|  | Baseline | 164 | 59.1 [56.4 to 61.7] | 47 | 65.3 [60.3 to 70.2] |  |  |  |
|  | Follow-up | 132 | 60.0 [57.2 to 62.8] | 36 | 64.3 [58.9 to 69.6] | 157 | 1.9 [-3.1 to 6.9] | .462 |
|  |  |  | **n (%)** |  | **n (%)** |  |  |  |
| a. Self-management (PAM-13): This scale ranges from 0 to 100, where higher scores indicate greater activation. Change is reported as the change in total score, mean (SD). b. Self-management levels (PAM levels): The levels are categorised as follows: Level 1 is ≤47.0, Level 2 is 47.1-55.1, Level 3 is 55.2-67.0, and Level 4 is ≥67.1. Higher level indicate higher self-management. Change is reported as the number (%) of participants who moved up one or more levels from baseline to follow-up. c. HbA_1c_: The treatment goal for HbA1c in Norwegian people with type 1 diabetes is 53 mmol/mol, and values ≥75 mmol/mol are considered poor glycaemic control. A change in HbA_1c_ of 5.5 mmol/mol is considered clinically relevant. d. WHO-5: This scale ranges from 0 to 100, where higher scores indicate better well-being. Scores below 50 indicate mild to severe depressive symptoms. Change is reported as the change in total score, mean (SD). e. WHO-5 score <50: Refers to the number of participants with scores below 50, indicating depressive symptoms. Change is reported as the number (%) of participants who improved from a score below 50 at baseline to a score above 50 at follow-up. f. MD: Estimated mean between-group difference. | | | | | | | | |

| Supplement 5B: Results from the GLM analyses for continuous and categorical outcomes between DigiDiaS care and usual care on clinical outcomes (initial group choice analysis). | | | | | | | | |
| --- | --- | --- | --- | --- | --- | --- | --- | --- |
|  |  | **DigiDiaS care** | | **Usual care** | | **Between groups** | | |
|  |  | **N** | **n (%)** | **N** | **n (%)** | **N** | **MD^b^ [95% CI]** | ***P*** |
| **Insulin delivery, pump** | | | | | | | | |
|  | Baseline | 185 | 74 (40.0) | 52 | 12 (23.1) |  |  |  |
|  | Follow-up | 176 | 90 (48.6) | 49 | 8 (15.4) |  |  |  |
|  | Change in level | 176 | 24 (13.6) | 49 | 2 (4.1) |  |  | N/A |
| **Blood glucose monitoring, CGM** | | | | | | | | |
|  | Baseline | 184 | 178 (96.2) | 52 | 47 (90.4) |  |  |  |
|  | Follow-up | 176 | 173 (93.5) | 49 | 46 (88.5) |  |  |  |
|  | Change in level | 175 | 3 (1.7) | 49 | 2 (4.1) |  |  | N/A |
| **Late complications from diabetes** | | | | | | | | |
|  | Baseline | 185 | 78 (42.2) | 52 | 23 (44.2) |  |  |  |
|  | Follow-up | 175 | 76 (41.1) | 47 | 24 (46.2) |  |  |  |
|  | |  | **Estimated mean**  **[95% CI]** |  | **Estimated mean**  **[95% CI]** |  |  |  |
| **LDL-cholesterol^a^** | | | | | | | | |
|  | Baseline | 183 | 2.6 [2.5 to 2.7] | 52 | 2.3 [2.1 to 2.5] |  |  |  |
|  | Follow-up | 98 | 2.4 [2.3 to 2.6] | 20 | 2.1 [1.7 to 2.4] | 117 | 0.1 [-0.3 to 0.5] | .601 |
| **Blood pressure systolic, mmHg** | | | | | | | | |
|  | Baseline | 172 | 131.5  [129.1 to 133.8] | 47 | 134.7  [130.2 to 139.2] |  |  |  |
|  | Follow-up | 74 | 132.0  [128.6 to 135.4] | 17 | 132.6  [125.6 to 139.7] | 86 | 2.56  [-5.6 to 10.7] | .537 |
| **Blood pressure diastolic, mmHg** | | | | | | | | |
|  | Baseline | 172 | 79.4 [78.1 to 80.6] | 47 | 78.2 [75.8 to 80.6] |  |  |  |
|  | Follow-up | 74 | 80.3 [78.4 to 82.1] | 17 | 78.8 [75.0 to 82.6] | 86 | 0.26 [-4.2 to 4.7] | .907 |
| a. LDL-cholesterol: Statin therapy is recommended for all people with diabetes aged 40-80 years without known cardiovascular disease if LDL cholesterol exceeds 2.5 mmol/mol or if overall cardiovascular risk is high. b. MD: Estimated mean between-group difference. | | | | | | | | |

| Supplement 5C: Self-reported outcomes: Results from the GLM analyses for continuous and categorical outcomes between DigiDiaS care and usual care on diabetes distress, health literacy and experience of involvement (initial group choice analysis). | | | | | | | | |
| --- | --- | --- | --- | --- | --- | --- | --- | --- |
|  | | **DigiDiaS care** | | **Usual care** | | **Between groups** | | |
|  | | **N** | **Estimated mean**  **[95% CI]** | **N** | **Estimated mean**  **[95% CI]** | **N** | **MD^g^**  **[95% CI]** | ***P*** |
| **Diabetes distress score (PAID)^a^** | | | | | | | | |
|  | Baseline | 164 | 25.9 [23.4 to 28.4] | 47 | 22.3 [17.6 to 27.0] |  |  |  |
|  | Follow-up | 129 | 24.6 [21.9 to 27.3] | 37 | 19.4 [14.3 to 24.4] | 155 | 1.6 [-3.3 to 6.5] | .517 |
|  | |  | **n (%)** |  | **n (%)** |  |  |  |
| **PAID score > 40^b^** | | | | | | | | |
|  | Baseline, n (%) | 164 | 35 (18.9) | 47 | 7 (13.5) |  |  |  |
|  | Follow-up, n (%) | 129 | 27 (14.6) | 37 | 5 (9.6) |  |  |  |
|  | Change in level | 120 | 12 (10.0) | 35 | 2 (5.7) |  |  | N/A |
|  |  |  | **Estimated mean**  **[95% CI]** |  | **Estimated mean**  **[95% CI]** |  |  |  |
| **Health literacy score (HLS19-Q12)^c^** | | | | | | | | |
|  | Baseline | 164 | 33.9 [32.9 to 34.9] | 47 | 34.0 [32.1 to 35.9] |  |  |  |
|  | Follow-up | 132 | 35.0 [33.9 to 36.1] | 37 | 34.4 [32.3 to 36.4] | 158 | 0.69 [-1.7 to 3.1] | .569 |
| **Healthcare score (HC)^d^** | | | | | | | | |
|  | Baseline | 164 | 11.7 [11.3 to 12.1] | 47 | 11.2 [10.5 to 12.0] |  |  |  |
|  | Follow-up | 132 | 12.0 [11.5 to 12.4] | 37 | 11.4 [10.5 to 12.2] | 158 | 0.21 [-0.8 to 1.3] | .697 |
| **Disease prevention score (DP)^d^** | | | | | | | | |
|  | Baseline | 164 | 10.2 [9.7 to 10.6] | 47 | 10.8 [9.9 to 11.7] |  |  |  |
|  | Follow-up | 132 | 10.7 [10.2 to 11.2] | 37 | 10.9 [10.0 to 11.9] | 158 | 0.37 [-0.7 to 1.5] | .513 |
| **Health promotion score (HP)^d^** | | | | | | | | |
|  | Baseline | 164 | 12.1 [11.7 to 12.4] | 47 | 12.0 [11.3 to 12.6] |  |  |  |
|  | Follow-up | 132 | 12.3 [11.9 to 12.6] | 37 | 12.0 [11.3 to 12.8] | 158 | 0.13 [-0.7 to 1.0] | .763 |
|  |  |  | **n (%)** |  | **n (%)** |  |  |  |
| **Health literacy levels^e^** | | | | | | | | |
|  | Below level 1 |  |  |  |  |  |  |  |
|  | Baseline, n (%) | 164 | 23 (12.4) | 47 | 4 (7.7) |  |  |  |
|  | Follow-up, n (%) | 132 | 15 (8.1) | 37 | 3 (5.8) |  |  |  |
|  | Level 1 |  |  |  |  |  |  |  |
|  | Baseline, n (%) | 164 | 38 (20.5) | 47 | 14 (26.9) |  |  |  |
|  | Follow-up, n (%) | 132 | 32 (17.3) | 37 | 13 (25.0) |  |  |  |
|  | Level 2 |  |  |  |  |  |  |  |
|  | Baseline, n (%) | 164 | 62 (33.5) | 47 | 17 (32.7) |  |  |  |
|  | Follow-up, n (%) | 132 | 43 (23.2) | 37 | 8 (15.4) |  |  |  |
|  | Level 3 |  |  |  |  |  |  |  |
|  | Baseline, n (%) | 164 | 41 (22.2) | 47 | 12 (23.1) |  |  |  |
|  | Follow-up, n (%) | 132 | 42 (22.7) | 37 | 13 (25.0) |  |  |  |
|  | Change in level | 122 | 36 (30.3) | 35 | 12 (34.2) |  |  | .569 |
|  |  |  | **Estimated mean**  **[95% CI]** |  | **Estimated mean**  **[95% CI]** |  |  |  |
| **Experience of involvement score ^f^** | | | | | | | | |
|  | Baseline | 163 | 19.4 [18.7 to 20.0] | 47 | 17.8 [16.7 to 19.0] |  |  |  |
|  | Follow-up | 132 | 19.3 [18.6 to 20.0] | 37 | 17.8 [16.5 to 19.1] | 158 | -0.07 [-1.6 to 1.4] | .920 |
| The number of respondents for self-reported data may vary due to incomplete questionnaires and dropout from baseline to follow-up. a. Problem Areas in Diabetes (PAID): This scale ranges from 0 to 100, with higher scores indicating greater emotional distress related to diabetes. A score of 40 or higher suggests severe emotional distress. Change is reported as the change in total score, mean (SD). b. Diabetes distress (PAID score >40): Refers to the number of participants with scores of 40 or higher, indicating diabetes-related emotional distress. Change is reported as the number (%) of participants who improved from a score above 40 at baseline to a score below 40 at follow-up. c. Health literacy scale (HLS19-Q12): The overall scale ranges from 12 to 48, where higher scores indicate better health literacy.  d. Health literacy domains: Scores for each domain range from a minimum of 0 to a maximum of 16. Change is reported as the change in total score, mean (SD), for both the overall scale and the individual domains. e. Health literacy levels: Levels are defined as follows: Level 1 is 27 points or above, Level 2 is 33 points or above, and Level 3 is 39 points or above, each with specific characteristics. Higher level indicates higher health literacy. Change is reported as the number (%) of participants who moved up one or more levels from baseline to follow-up. f. Experience of involvement: This scale ranges from 0 to 25, with higher scores indicate better patient participation. Change is reported as the change in total score, mean (SD). g. MD: Estimated mean between-group difference. | | | | | | | | |
