# Supplementary material for: Digital Outpatient Care for Patients With Type 1 Diabetes (DigiDiaS): Pragmatic Observational Pre-Post Study
Source: J Med Internet Res. 2026 Jul 13;28:e94782. doi: 10.2196/94782 (PMC13408466; doi:10.2196/94782)
Supplement: Multimedia Appendix 6 [file jmir_v28i1e94782_app6.docx]

### Supplement 6: Initial group choice: Extreme-case sensitivity analysis in primary outcome

Supplement 6: Extreme‑case sensitivity analyses in which missing primary‑outcome values were replaced with the lowest (worst case) or the highest (best case) observed value on initial group choice is shown in Supplement 6A and as‑treated in Supplement 6B.

| Supplement 6A: Initial group choice extreme-case sensitivity analysis in primary outcome | | | | | | | | |
| --- | --- | --- | --- | --- | --- | --- | --- | --- |
|  |  | **DigiDiaS care** | | **Usual care** | | **Between groups** | |  |
| **Primary outcome** | | | | | | | | |
|  | | **N** | **Estimated mean**  **[95% CI]** | **N** | **Estimated mean**  **[95% CI]** | **N** | **MD^c^**  **[95% CI]** | ***P*** |
| **Self-management score worst case (PAM-13)^a^** | | | | | | | | |
|  | Baseline | 164 | 71.2 [67.6 to 74.8] | 47 | 70.6 [63.7 to 77.4] |  |  |  |
|  | Follow-up | 131 | 55.5 [51.9 to 59.1] | 37 | 53.6 [46.7 to 60.4] | 157 | 1.3 [-9.3 to 11.8] | .812 |
| **Self-management score best case (PAM-13)^b^** | | | | | | | | |
|  | Baseline | 164 | 70.9 [68.4 to 73.4] | 47 | 71.6 [66.8 to 76.4] |  |  |  |
|  | Follow-up | 131 | 78.5 [76.0 to 81.0] | 37 | 77.8 [73.0 to 82.6] | 157 | 1.3 [-5.5 to 8.2] | .701 |
| a - worst‑case, replacing all missing follow‑up scores with the minimum possible value.  b - best‑case, replacing all missing follow‑up scores with the maximum possible value.  c - MD: Estimated mean between-group difference. | | | | | | | | |

| Supplement 6B: As-treated extreme-case sensitivity analysis in primary outcome | | | | | | | | |
| --- | --- | --- | --- | --- | --- | --- | --- | --- |
|  |  | **DigiDiaS care** | | **Usual care** | | **Between groups** | |  |
| **Primary outcome** | | | | | | | | |
|  | | **N** | **Estimated mean**  **[95% CI]** | **N** | **Estimated mean**  **[95% CI]** | **N** | **MD^c^**  **[95% CI]** | ***P*** |
| **Self-management score worst case (PAM-13)^a^** | | | | | | | | |
|  | Baseline |  | 70.8 [67.4 to 74.2] |  | 72.7 [64.4 to 81.0] |  |  |  |
|  | Follow-up |  | 54.8 [51.4 to 58.2] |  | 56.4 [48.1 to 64.8] |  | 0.3 [-12.1 to 12.7] | .964 |
| **Self-management score best case (PAM-13)^b^** | | | | | | | | |
|  | Baseline |  | 70.7 [68.3 to 73.1] |  | 73.2 [67.4 to 79.0] |  |  |  |
|  | Follow-up |  | 78.5 [76.1 to 80.8] |  | 77.5 [71.7 to 83.3] |  | 3.5 [-4.6 to 11.6] | .397 |
| a - worst‑case, replacing all missing follow‑up scores with the minimum possible value.  b - best‑case, replacing all missing follow‑up scores with the maximum possible value.  c - MD: Estimated mean between-group difference. | | | | | | | | |
