# Supplementary material for: Digital Outpatient Care for Patients With Type 1 Diabetes (DigiDiaS): Pragmatic Observational Pre-Post Study
Source: J Med Internet Res. 2026 Jul 13;28:e94782. doi: 10.2196/94782 (PMC13408466; doi:10.2196/94782)
Supplement: Multimedia Appendix 7 [file jmir_v28i1e94782_app7.docx]

### Supplement 7: As-treated: Use of features in DigiDiaS care

Supplement 7: Equivalent to Table 2 in the manuscript: Use of the features in the DigiDiaS care app with initial group choice distribution

|  |  | DigiDiaS care  n = 196 |
| --- | --- | --- |
| **Overall patient use** | |  |
|  | One or more messages sent, n (%) | 110 (56.1) |
|  | Received one or more PRO questionnaires, n (%) | 123 (62.8) |
|  | Completed one or more PRO questionnaires, n (%) | 93 (47.4) |
|  | Non-users, n (%) | 34 (17.3) |
| **PRO-based questionnaires** | |  |
|  | One or more PRO questionnaires received, n (%) | 172 (100) |
|  | One or more PRO questionnaires completed, n (%) | 100 (58.1) |
| **Message platform** | |  |
|  | Message sent from participant to clinic, sum (median; min-max) | 594 (1; 0-57) |
|  | Message sent from clinic to participant, sum (median; min-max) | 544 (1; 0-37) |
| **Individually tailored information section** | |  |
|  | Information section in use, n (%) | 61 (31.1) |
| **Re-consent** | |  |
|  | Re-consenting to app use, n (%) | 110 (56.1) |
