# Supplementary material for: Digital Outpatient Care for Patients With Type 1 Diabetes (DigiDiaS): Pragmatic Observational Pre-Post Study
Source: J Med Internet Res. 2026 Jul 13;28:e94782. doi: 10.2196/94782 (PMC13408466; doi:10.2196/94782)
Supplement: Multimedia Appendix 8 [file jmir_v28i1e94782_app8.docx]

### Supplement 8: As-treated: boxplot for messages sent from patients

Supplement 8: Equivalent to Figure 3 in the manuscript. Boxplot of number of messages sent from patients to the healthcare service initial group choice distribution


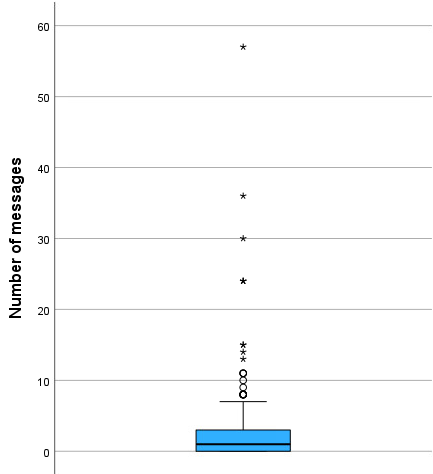

Circles (o): Mild outliers, which fall between 1.5 and 3 times the interquartile range from the box.
Stars (*): Extreme outliers, which fall more than 3 times the interquartile range from the box.
