# Supplementary material for: Digital Outpatient Care for Patients With Type 1 Diabetes (DigiDiaS): Pragmatic Observational Pre-Post Study
Source: J Med Internet Res. 2026 Jul 13;28:e94782. doi: 10.2196/94782 (PMC13408466; doi:10.2196/94782)
Supplement: Multimedia Appendix 9 [file jmir_v28i1e94782_app9.docx]

### Supplement 9: As-treated: unadjusted analysis: change from baseline to follow-up primary and secondary outcomes

Supplement 9: As treated analysis on between-group change from baseline to follow-up on primary and secondary outcomes (Supplement 9A), disease specific variables (Supplement 9B) and self-reported (Supplement 9C)

Supplement 9A: Equivalent to Table 3 in the manuscript: Change from baseline to follow-up between DigiDiaS care and usual care on clinical outcomes with as-treated distribution

|  |  | **DigiDiaS care** | | **Usual care** | | **Between groups** | |  |
| --- | --- | --- | --- | --- | --- | --- | --- | --- |
|  | | **N** | **Estimated mean [95 % CI]** | **N** | **Estimated mean [95 % CI]** | **N** | **MD [95% CI]** | ***P*** |
| **Self-management score (PAM-13)^a^** | | | | | | | | |
|  | Baseline | 180 | 70.7 [68.5 to 72.9] | 31 | 73.5 [68.3 to 78.6] |  |  |  |
|  | Follow-up | 143 | 71.2 [68.8 to 73.6] | 25 | 71.8 [66.1 to 77.6] | 157 | 2.1 [-5.4 to 9.6] | .585 |
|  | |  | **n (%)** |  | **n (%)** |  |  |  |
| **Self-management levels (PAM-13)^b^** | | | | | | | | |
|  | Level 1 |  |  |  |  |  |  |  |
|  | Baseline | 180 | 13 (7.2) | 31 | 1 (3.2) |  |  |  |
|  | Follow-up | 143 | 6 (4.2) | 25 | 2 (8) |  |  |  |
|  | Level 2 |  |  |  |  |  |  |  |
|  | Baseline | 180 | 10 (5.6) | 31 | 3 (9.7) |  |  |  |
|  | Follow-up | 143 | 10 (7.0) | 25 | 1 |  |  |  |
|  | Level 3 |  |  |  |  |  |  |  |
|  | Baseline | 180 | 62 (34.4) | 31 | 6 (19.4) |  |  |  |
|  | Follow-up | 143 | 28 (19.6) | 25 | 7 (28.0) |  |  |  |
|  | Level 4 |  |  |  |  |  |  |  |
|  | Baseline | 180 | 95 (52.8) | 31 | 21 (67.7) |  |  |  |
|  | Follow-up | 143 | 99 (69.2) | 25 | 15 (60.0) |  |  |  |
|  | Change in level | 133 | 26 (19.5) | 24 | 7 (29.2) |  |  | .430 |
|  | |  | **Estimated mean [95 % CI]** |  | **Estimated mean [95 % CI]** |  |  |  |
| **HbA_1c_^b^** | | | | | | | | |
|  | Baseline | 202 | 60.8 [59.0 to 62.7] | 33 | 52.6 [48.0 to 57.3] |  |  |  |
|  | Follow-up | 145 | 57.8 [55.7 to 60.0] | 20 | 54.5 [49.0 to 60.0] | 146 | -4.9 [-10.2 to 0.5] | .076 |
|  | |  | **n (%)** |  | **n (%)** |  |  |  |
| **HbA_1c_ above 75 mmol/mol** | | | | | | | | |
|  | Baseline | 202 | 36 (17.8) | 33 | 0 |  |  |  |
|  | Follow-up | 145 | 17 (11.7) | 20 | 0 |  |  |  |
|  | Change | 145 | 18 (12.4) | 19 | 0 |  |  | N/A |
|  |  |  | **Estimated mean**  **[95 % CI]** |  | **Estimated mean [95 % CI]** |  |  |  |
| **Time in range** | | | | | | | | |
|  | Baseline | 167 | 60.4 [57.6 to 63.2] | 26 | 66.0 [58.8 to 73.1] |  |  |  |
|  | Follow-up | 138 | 62.7 [59.7 to 65.8] | 18 | 60.4 [52.1 to 68.6] | 134 | 7.9 [-1.1 to 16.9] | .085 |
| **Well-being (WHO-5)^d^** | | | | | | | |  |
|  | Baseline | 180 | 59.8 [57.3 to 62.4] | 31 | 64.0 [57.8 to 70.1] |  |  |  |
|  | Follow-up | 145 | 60.4 [57.7 to 63.1] | 23 | 64.0 [57.2 to 70.6] | 157 | 0.55 [-5.3 to 6.5] | .854 |
|  | |  | **n (%)** |  | **n (%)** |  |  |  |
| **Well-being score <50^e^** | | | | | | | | |
|  | Baseline | 180 | 42 (23.3) | 31 | 4 (12.9) |  |  |  |
|  | Follow-up | 145 | 41 (28.3) | 23 | 3 (13.0) |  |  |  |
|  | Change | 135 | 6 (4.4) | 22 | 2 (6.1) |  |  | N/A |
| a. Self-management (PAM-13): This scale ranges from 0 to 100, where higher scores indicate greater activation. Change is reported as the change in total score, mean (SD). b. Self-management levels (PAM levels): The levels are categorised as follows: Level 1 is ≤47, Level 2 is 47.1–55.1, Level 3 is 55.2–67, and Level 4 is ≥67.1. Change is reported as the number (%) of participants who moved up one or more levels from baseline to follow-up. c. HbA_1c_: Treatment goal for HbA1c in Norwegian participants with type 1 diabetes is 53 mmol/mol and 75 mmol/mol is considered poor regulated diabetes. Change in HbA_1c_ of 5.5 mmol/mol is considered clinically relevant. d. WHO-5: This scale ranges from 0 to 100, where higher scores indicate better well-being. Scores below 50 indicates mild to severe depressive symptoms. Change is reported as the change in total score, mean (SD). e. WHO score < 50: Refers to the number of participants with scores below 50, which indicates depressive symptoms. Change is reported as the number (%) of participants who improved from a score below 50 at baseline to a score above 50 at follow-up. | | | | | | | | |

Supplement 9B: Results from the GLM analyses for continuous and categorical outcomes between DigiDiaS care and usual care on clinical outcomes

|  |  | **DigiDiaS care** | | **Usual care** | | **Between groups** | |  |
| --- | --- | --- | --- | --- | --- | --- | --- | --- |
|  | | **N** | **n (%)** | **N** | **n (%)** | N | **MD [95% CI]** | ***P*** |
| **Inulin delivery, pump** | |  |  |  |  |  |  |  |
|  | Baseline | 203 | 79 (38.9) | 34 | 7 (20.6) |  |  |  |
|  | Follow-up | 194 | 95 (49.0) | 31 | 3 (9.7) |  |  |  |
|  | Change | 194 | 26 (13.4) | 31 | 0 |  |  | N/A |
| **Blood glucose monitoring, CGM** | | | | | | | | |
|  | Baseline | 202 | 194 (96.0) | 31 | 31 (91.2) |  |  |  |
|  | Follow-up | 194 | 191 (98.5) | 31 | 28 (90.3) |  |  |  |
|  | Change | 193 | 5 (2.6) | 31 | 0 |  |  | N/A |
| **Late complications from diabetes** | | | | | | | | |
|  | Baseline | 203 | 85 (41.9) | 34 | 16 (47.1) |  |  |  |
|  | Follow-up | 191 | 85 (44.5) | 31 | 15 (48.4) |  |  |  |
|  |  |  | **Estimated mean**  **[95 % CI]** |  | **Estimated mean**  **[95 % CI]** |  |  |  |
| **LDL-cholesterol^b^** | | | | | | | | |
|  | Baseline | 201 | 2.5 [2.4 to 2.67] | 34 | 2.3 [2.01 to 2.57] |  |  |  |
|  | Follow-up | 105 | 2.4 [2.2 to 2.5] | 13 | 2.2 [1.7 to 2.6] | 117 | -0.6 [-0.51 to 0.39] | .791 |
| **Blood pressure systolic, mmHg** | | | | | | | | |
|  | Baseline | 188 | 131.2 [129.0 to 133.4] | 31 | 138.0 [132.3 to 143.4] |  |  |  |
|  | Follow-up | 78 | 132.1 [128.8 to 135.4] | 13 | 132.4 [124.3 to 140.5] | 86 | 6.3 [-2.66 to 15.39] | .167 |
| **Blood pressure diastolic, mmHg** | | | | | | | | |
|  | Baseline | 188 | 79.0 [77.7 to 80.1] | 31 | 80.2 [77.3 to 83.2] |  |  |  |
|  | Follow-up | 78 | 80.1 [78.4 to 82.0] | 13 | 78.8 [74.4 to 83.1] | 86 | 2.7 [-2.2 to 7.6] | .289 |
| a. LDL-cholesterol: Statin therapy is recommended for all people with diabetes aged 40 - 80 years without known cardiovascular disease if LDL cholesterol exceeds 2.5 mmol/mol or when overall risk is high. | | | | | | | | |

| Supplement 9C: Results from the GLM analyses for continuous and categorical outcomes between DigiDiaS care and usual care on diabetes distress, health literacy and experience of involvement . | | | | | | | | |
| --- | --- | --- | --- | --- | --- | --- | --- | --- |
|  | | **DigiDiaS care** | | **Usual care** | | **Between groups** | | |
|  | | **N** | **Estimated mean [95 % CI]** | **N** | **Estimated mean [95 % CI]** | **N** | **MD [95% CI]** | ***P*** |
| **Diabetes distress score (PAID)^a^** | | | | | | | | |
|  | Baseline | 180 | 25.6 [23.3 to 28.1] | 31 | 21.6 [15.8 to 27.5] |  |  |  |
|  | Follow-up | 145 | 24.1 [21.5 to 26.6] | 24 | 19.4 [13.2 to 25.7] | 155 | 0.66 [-5.1 to 6.4] | .820 |
|  |  |  | **n (%)** |  | **n (%)** |  |  |  |
| **PAID score > 40^b^** | | | | | | | | |
|  | Baseline | 180 | 38 (21.1) | 31 | 4 (12.9) |  |  |  |
|  | Follow-up | 142 | 28 (19.7) | 24 | 4 (16.7) |  |  |  |
|  | Change | 132 | 12 (9.1) | 23 | 2 (8.7) |  |  | N/A |
|  |  |  | **Estimated mean**  **[95 % CI]** |  | **Estimated mean**  **[95 % CI]** |  |  |  |
| **Health literacy score^c^** | | | | | | | | |
|  | Baseline | 180 | 34.1 [33.1 to 35.0] | 31 | 33.0 [30.6 to 35.3] |  |  |  |
|  | Follow-up | 145 | 35.1 [34.1 to 36.1] | 24 | 33.0 [30.4 to 35.5] | 158 | 1.1 [-1.7 to 3.9] | .447 |
| **Healthcare (HC)^d^** | | | | | | | | |
|  | Baseline | 180 | 11.6 [11.2 to 12.1] | 31 | 11.0 [10.1 to 12.0] |  |  |  |
|  | Follow-up | 145 | 12.1 [11.5 to 12.4] | 24 | 10.8 [9.8 to 12.0] | 158 | 0.4 [-0.8 to 1.6] | .498 |
| **Disease prevention (DP)^d^** | | | | | | | | |
|  | Baseline | 180 | 10.3 [9.8 to 10.7] | 31 | 10.2 [9.1 to 11.3] |  |  |  |
|  | Follow-up | 145 | 10.8 [10.3 to 11.3] | 24 | 10.3 [9.1 to 11.6] | 158 | 0.3 [-1.0 to 1.6] | .634 |
| **Health promotion (HP)^d^** | | | | | | | |  |
|  | Baseline | 180 | 12.1 [11.7 to 12.4] | 31 | 11.8 [11.1 to 12.6] |  |  |  |
|  | Follow-up | 145 | 12.2 [12.0 to 12.6] | 24 | 11.6 [10.7 to 12.5] | 158 | 0.3 [-0.6 to 1.3] | .479 |
|  | |  | **n (%)** |  | **n (%)** |  |  |  |
| **Health literacy levels^e^** | | | | | | | | |
|  | Below level 1 |  |  |  |  |  |  |  |
|  | Baseline | 180 | 23 (12.8) | 31 | 4 (12.9) |  |  |  |
|  | Follow-up | 145 | 15 (10.3) | 24 | 3 (12.5) |  |  |  |
|  | Level 1 |  |  |  |  |  |  |  |
|  | Baseline | 180 | 43 (23.9) | 31 | 9 (29.0) |  |  |  |
|  | Follow-up | 145 | 35 (24.1) | 24 | 10 (41.7) |  |  |  |
|  | Level 2 |  |  |  |  |  |  |  |
|  | Baseline | 180 | 67 (37.2) | 31 | 12 (38.7) |  |  |  |
|  | Follow-up | 145 | 47 (32.4) | 24 | 4 (16.7) |  |  |  |
|  | Level 3 |  |  |  |  |  |  |  |
|  | Baseline | 180 | 47 (26.1) | 31 | 6 (19.4) |  |  |  |
|  | Follow-up | 145 | 48 (33.1) | 24 | 7 (29.2) |  |  |  |
|  | Change in level | 135 | 41 (30.4) | 23 | 7 (30.4) |  |  | .995 |
|  |  |  | **Estimated mean [95 % CI]** |  | **Estimated mean [95 % CI]** |  |  |  |
| **Experience of involvement score ^i^** | | | | | | | | |
|  | Baseline | 179 | 19.3 [18.6 to 19.9] | 31 | 17.4 [16.1 to 19.0] |  |  |  |
|  | Follow-up | 145 | 19.2 [18.6 to 20.0] | 24 | 17.1 [15.5 to 18.7] | 158 | 0.3 [-1.4 to 2.1] | .732 |
| The number of respondents for self-reported data may vary due to incomplete questionnaires and dropout from baseline to follow-up. a. Problem Areas in Diabetes (PAID): This scale ranges from 0 to 100, with higher scores indicating greater emotional distress related to diabetes. A score of 40 or higher suggests severe emotional distress. Change is reported as the change in total score, mean (SD). b. Diabetes distress (PAID score > 40): Refers to the number of participants with scores of 40 or higher, indicating diabetes-related emotional distress. Change is reported as the number (%) of participants who improved from a score above 40 at baseline to a score below 40 at follow-up. c. Health Literacy scale (HLS19-Q12): The overall scale ranges from 12 to 48, where higher scores indicate better health literacy.  d. Health literacy domains: Scores for each domain range from a minimum of 0 to a maximum of 16. Change is reported as the change in total score, mean (SD), for both the overall scale and the individual domains. e. Health Literacy levels: Levels are defined as follows: Level 1 is 27 points or above, Level 2 is 33 points or above, and Level 3 is 39 points or above, each with specific characteristics. Change is reported as the number (%) of participants who moved up one or more levels from baseline to follow-up. f. Experience of involvement: This scale ranges from 0 to 25, with higher scores indicating better patient participation. Change is reported as the change in total score, mean (SD). MD: estimated mean between group difference | | | | | | | | |
