# Supplementary material for: Digital Outpatient Care for Patients With Type 1 Diabetes (DigiDiaS): Pragmatic Observational Pre-Post Study
Source: J Med Internet Res. 2026 Jul 13;28:e94782. doi: 10.2196/94782 (PMC13408466; doi:10.2196/94782)
Supplement: Multimedia Appendix 10 [file jmir_v28i1e94782_app10.docx]

### Supplement 10: As-treated: health care use

Equivalent to Table 4 in the manuscript: Healthcare utilisation of the DigiDiaS care and usual care group

|  |  | **DigiDiaS care**  **(n = 196)** | **Usual care**  **(n = 32)** | **Between group *P* value** |
| --- | --- | --- | --- | --- |
| **Overall healthcare utilisation** | |  |  |  |
|  | Individual consultation attendance last year, n (%) | 176 (89.8) | 25 (78.1) | .041 |
|  | Group education participation last year, n (%) | 58 (29.6) | 1 | N/A |
|  | No consultation or course engagement, n (%) | 15 (7.7) | 7 (22.6) | N/A |
| **Individual consultation, sum** | | **310** | **38** | **.041** |
|  | Diabetes specialist nurse, sum (%) | 212 (68.4) | 23 (60.5) | .006 |
|  | Endocrinologist, sum (%) | 78 (25.2) | 14 (36.8) | .543 |
|  | Clinical nutritionist, sum (%) | 13 (4.2) | 1 | N/A |
|  | Consultations triggered by messages from participant, sum (%) | 7 (2.3) | N/A | N/A |
| **Mode of individual consultation** | |  |  |  |
|  | In person, sum (%) | 233 (75.2) | 29 (76.3) | .002 |
|  | Telephone, sum (%) | 63 (20.3) | 9 (23.1) | N/A |
|  | Video, sum (%) | 14 (4.5) | 0 | N/A |
| **Group education, sum** | | **71** | **1** | N/A |
|  | Diabetes education course, sum (%) | 7 (9.9) | 1 | N/A |
|  | Carbohydrate counting, sum (%) | 32 (45.1) | 0 | N/A |
|  | Pump, sum (%) | 32 (45.1) | 0 | N/A |
| **Change of appointment, sum** | | **98** | **11** | **.200** |
|  | Patient change/cancellation of individual consultation or group education, sum (%) | 69 (70.4) | 8 (72.7) | N/A |
|  | The clinic has changed an appointment, sum (%) | 29 (29.6) | 3 (27.3) | N/A |
| **Non-attendance, sum** | | **18** | **1** | **N/A** |
|  | Non-attendance individual consultation, sum (%) | 13 (72.2) | 1 | N/A |
|  | Non-attendances at group education, sum (%) | 5 (27.8) | 0 | N/A |
| Percentage is based on the number above in bold.  N/A – Statistical analysis not applicable due to small number The number of respondents for self-reported data may vary due to incomplete questionnaires and dropout from baseline to follow-up. | | | | |
