# Supplementary material for: Digital Outpatient Care for Patients With Type 1 Diabetes (DigiDiaS): Pragmatic Observational Pre-Post Study
Source: J Med Internet Res. 2026 Jul 13;28:e94782. doi: 10.2196/94782 (PMC13408466; doi:10.2196/94782)
Supplement: Multimedia Appendix 11 [file jmir_v28i1e94782_app11.docx]

### Supplement 11: Initial group choice: responders and nonresponders

To assess potential responder and non‑responder bias, selected characteristics were compared between participants grouped by their response status to the self‑reported outcome questionnaires, according to initial group choice allocation within the DigiDiaS care and Usual care groups. Responders were defined as participants who completed the questionnaire at baseline and follow‑up, non‑responders were those who completed the self-reported questionnaire at baseline and not at follow-up. Categorical variables were compared using chi‑squared tests, and continuous variables using Mann–Whitney U tests. Counts for participants who did not respond at either time point (non-responders at all) are reported but were not included in the comparative analyses.

Supplement 11: Responders and non-responders analysis by initial group choice for DigiDiaS care (Supplement 11A) and usual care (Supplement 11B).

Supplement 11A: Selected characteristics by response status in the DigiDiaS care group (initial group choice allocation)

| Variable | | **Responders**  **n= 123** | **Non-responders**  **n= 50** | ***P^a^*** | **Non-responders at all**  **n= 12** |
| --- | --- | --- | --- | --- | --- |
| **Gender, woman, n (%)** | | 63 (51.2) | 24 (48.0) | .701 | 4 (33.3) |
| **Age, years, median (min-max)** | | 48 (19;79) | 44.5 (19;69) | .058 | 42 (19;65) |
| **Diabetes duration, years, median (min-max)** | | 19 (0;49) | 13.5 (0;59) | .036 | 21 (9;56) |
| **HbA1c,** measured at follow-up in **mmol/mol, median (min-max)** | | 55.0 (30;87) | 55.5 (34;146) | .645 | 68 (57;96) |
|  | Missing, n (%) | 30 (24.4) | 18 (36) |  | 3 (25) |
| **Late-complications from diabetes, measured at follow-up,** one or more**, n (%)** | | 56 (45.5) | 14 (28.0) | .088 | 6 (50) |
|  | Missing, n (%) | 3 (2.4) | 6 (12) |  | 1 (8.3) |

a - Comparisons are restricted to responders (completed baseline and follow‑up) versus non‑responders (completed baseline only). Participants with no response at either time point are reported for count only and were not included in the comparative analysis.

Supplement 11B: Selected characteristics by response status in the usual care group (initial group choice allocation)

| Variable | | **Respondes**  **n= 34** | **Non-responders**  **n= 15** | ***P^a^*** | **Non-responders at all**  **n= 3** |
| --- | --- | --- | --- | --- | --- |
| **Gender, woman, n (%)** | | 14 (41.2) | 3 (20.0) | N/A | 0 |
| **Age, years, median (min-max)** | | 61.5 (23;81) | 55 (25;76) | .379 | 59 (25;61) |
| **Diabetes duration, years, median (min-max)** | | 29 (2;58) | 25 (3;52) | .273 | 36 (6;39) |
| **HbA1c,** measured at follow-up in **mmol/mol, median (min-max)** | | 54 (39;71) | 55.5 (45;64) | .581 | 70 (68;72) |
|  | Missing, n (%) | 11 (32.4) | 9 (60) |  | 1 (33.3) |
| **Late-complications from diabetes, measured at follow-up,** one or more**, n (%)** | | 18 (52.9) | 5 (33.3) | N/A | 1 (33.3) |
|  | Missing, n (%) | 1 (2.9) | 4 (26.7) |  | 0 |

a - Comparisons are restricted to responders (completed baseline and follow‑up) versus non‑responders (completed baseline only). Participants with no response at either time point are reported for count only and were not included in the comparative analysis.
