# Supplementary material for: Digital Outpatient Care for Patients With Type 1 Diabetes (DigiDiaS): Pragmatic Observational Pre-Post Study
Source: J Med Internet Res. 2026 Jul 13;28:e94782. doi: 10.2196/94782 (PMC13408466; doi:10.2196/94782)
Supplement: Multimedia Appendix 12 [file jmir_v28i1e94782_app12.docx]

### Supplement 12: Initial group choice and as-treated: digital health literacy: the digital skills HLS19-DHC-NO_Norwegian

Supplement 12: Digital health literacy for initial group choice (Supplement 12A) and As-treated (Supplement 12B).

Suppelement 12 A: Initial group choice: Comparison of scoring very difficult and very easy between DigiDiaS care and usual care on items from digital skills in digital health literacy.

|  | DigiDiaS care  n = 131 | | | Usual care  n = 43 | | |  |
| --- | --- | --- | --- | --- | --- | --- | --- |
| On a scale from very difficult to very easy, how easy will it be for you to | Very difficult | Very easy | Missing | Very difficult | Very easy | Missing | *P* |
|  | n (%) | n (%) | n (%) | n (%) | n (%) | n (%) |  |
| Make a video call with a health care professional on a mobile phone or tablet? | 2 (1.5) | 53 (40.5) | 12 (9.1) | 3 (7.0) | 12 (2.3) | 14 (32.5) | .527 |
| Follow the advice from health care professionals during a video call? | 2 (1.5) | 53 (40.5) | 17 (13.0) | 3 (7.0) | 12 (2.3) | 16 (37.2) | .089 |
| contact healthcare professionals by phone when needed?^A^ | 6 (4.6) | 49 (37.4) | 4 (3.0) | 0 | 15 (34.9) | 11 (25.5) | .469 |
| use medical equipment to measure blood pressure or similar?^A^ | 0 | 65 (49.6) | 20 (15.2) | 0 | 13 (30.2) | 17 (39.5) | .730 |
| Use a mobile phone or tablet to record results from measurements you take yourself (e.g., blood pressure)? | 2 (1.5) | 54 (41.2) | 30 (23.0) | 2 (4.7) | 11 (25.6) | 20 (46.5) | .021 |
| use a mobile phone or tablet to monitor your measurements over time, e.g., assess whether blood pressure and blood glucose level are stable or have changed?^A^ | 2 (1.5) | 61 (46.6) | 14 (10.6) | 2 (4.7) | 13 (30.2) | 16 (37.2) | .277 |
| Provide feedback to health care professionals via apps or software on your mobile phone or tablet (e.g., confirm via text message that your blood pressure is now normal again)? | 2 (1.5) | 54 (41.2) | 28 (21.3) | 2 (4.7) | 9 (20.9) | 20 (46.5) | .155 |
| Complete e-learning courses to learn how to follow up and master your own illness? | 3 (2.3) | 40 (30.5) | 26 (19.8) | 2 (4.7) | 11 (25.6) | 17 (39.5) | .246 |
| Record your own health information in your personal health record on the internet (e.g., record use of medications, doctor visits, pain and the like)? | 3 (2.3) | 34 (26.0) | 29 (22.1) | 1 (2.3) | 9 (20.9) | 21 (48.8) | .444 |
| Communicate digitally with health care professionals regarding critical changes to your health? | 4 (3.1) | 37 (28.2) | 30 (23.0) | 2 (4.7) | 7 (16.3) | 19 (44.2) | .769 |
| ^A^ - Item 3, 4 and 6 had not previously been translated into English. We generated draft translations using AI and refined the wording to align with the phrasing of the other items to ensure consistency. | | | | | | | |

Supplement 12 B: As-treated distribution: Comparison of scoring very difficult and very easy between DigiDiaS care and usual care on items from digital skills in digital health literacy.

|  | DigiDiaS care  n = 144 | | | Usual care  n = 25 | | |  |
| --- | --- | --- | --- | --- | --- | --- | --- |
| On a scale from very difficult to very easy, how easy will it be for you to | Very difficult | Very easy | Missing | Very difficult | Very easy | Missing | *P* |
|  | n (%) | n (%) | n (%) | n (%) | n (%) | n (%) |  |
| Make a video call with a health care professional on a mobile phone or tablet? | 4 (2.8) | 61 (42.4) | 16 (11.1) | 2 (8) | 8 (32) | 5 (20) | .470 |
| Follow the advice from health care professionals during a video call? | 3 (2.1) | 57 (39.6) | 22 (15.2) | 2 (8) | 8 (32) | 6 (24) | .325 |
| contact healthcare professionals by phone when needed?^A^ | 6 (4.2) | 53 (36.8) | 8 (5.5) | 0 | 11 (44) | 2 (8) | .514 |
| use medical equipment to measure blood pressure or similar?^A^ | 0 | 70 (48.6) | 24 (16.6) | 0 | 9 (36) | 8 (32) | .625 |
| Use a mobile phone or tablet to record results from measurements you take yourself (e.g., blood pressure)? | 3 (2.1) | 56 (38.9) | 36 (25) | 1 (4) | 7 (28) | 9 (36) | .078 |
| use a mobile phone or tablet to monitor your measurements over time, e.g., assess whether blood pressure and blood glucose level are stable or have changed?^A^ | 3 (2.1) | 66 (45.8) | 18 (12.5) | 1 (4) | 8 (32) | 6 (24) | .678 |
| Provide feedback to health care professionals via apps or software on your mobile phone or tablet (e.g., confirm via text message that your blood pressure is now normal again)? | 3 (2.1) | 57 (39.6) | 34 (23.6) | 1 (4) | 6 (24) | 9 (36) | .431 |
| Complete e-learning courses to learn how to follow up and master your own illness? | 4 (2.8) | 45 (31.3) | 30 (20.8) | 1 (4) | 6 (24) | 8 (32) | .844 |
| Record your own health information in your personal health record on the internet (e.g., record use of medications, doctor visits, pain and the like)? | 3 (2.1) | 37 (25.7) | 36 (25) | 1 (4) | 6 (24) | 9 (36) | .791 |
| Communicate digitally with health care professionals regarding critical changes to your health? | 5 (3.5) | 39 (27.1) | 36 (25) | 1 (4) | 5 (20) | 8 (32) | .958 |
| ^A^ - Item 3, 4 and 6 had not previously been translated into English. We generated draft translations using AI and refined the wording to align with the phrasing of the other items to ensure consistency. | | | | | | | |
