# Supplementary material for: Digital Outpatient Care for Patients With Type 1 Diabetes (DigiDiaS): Pragmatic Observational Pre-Post Study
Source: J Med Internet Res. 2026 Jul 13;28:e94782. doi: 10.2196/94782 (PMC13408466; doi:10.2196/94782)
Supplement: Multimedia Appendix 13 [file jmir_v28i1e94782_app13.docx]

### Supplement 13: Initial group choice: GLM adjusted for covariates for other clinical and self-reported outcomes

Generalized linear models (GLM) adjusted covariates from baseline differences between DigiDiaS care and usual care (diabetes duration, insulin delivery method and WHO‑5 well‑being score) on initial group choice distribution.

|  |  | **DigiDiaS care** | | **Usual care** | | **Between groups** | |  |
| --- | --- | --- | --- | --- | --- | --- | --- | --- |
|  | | **N** | **Estimated mean**  **[95% CI]** | **N** | **Estimated mean**  **[95% CI]** | **N** | **MD [95% CI]** | ***P*** |
| **Self-management score (PAM-13)^a^** | | | | | | | | |
|  | Baseline | 164 | 71.1 [68.8 to 73.3] | 47 | 71.1 [66.8 to 75.3] |  |  |  |
|  | Follow-up | 131 | 71.2 [68.6 to 73.7] | 37 | 69.0 [64.1 to 73.8] | 157 | 0.87 [-4.1 to 8.6] | .482 |
| **HbA_1c_** | | | | | | | | |
|  | Baseline | 184 | 59.9 [57.8 to 62.0] | 51 | 54.3 [50.3 to 58.4] |  |  |  |
|  | Follow-up | 134 | 57.8 [55.5 to 60.2] | 51 | 53.4 [48.7 to 58.0] |  | -1.12 [-5.7 to 3.5] | .632 |
| **Time in range** | | | | | | | | |
|  | Baseline | 152 | 62.6 [59.7 to 65.6] | 41 | 64.5 [58.7 to 70.4] |  |  |  |
|  | Follow-up | 126 | 62.8 [59.5 to 66.1] | 30 | 62.0 [55.5 to 68.6] |  | 2.65 [-4.7 to 10.0] | .476 |
| **Well-being score (WHO-5)** | | | | | | | | |
|  | Baseline | 164 | 60.3 [59.0 to 61.7] | 47 | 61.2 [58.6 to 63.7] |  |  |  |
|  | Follow-up | 132 | 61.0 [59.4 to 62.5] | 36 | 59.4 [56.3 to 62.4] |  | 2.47 [-1.8 to 6.8] | .261 |
| **LDL-cholesterol** | | | | | | | | |
|  | Baseline | 183 | 2.6 [2.4 to 2.7] | 52 | 2.4 [2.2 to 2.7] |  |  |  |
|  | Follow-up | 98 | 2.4 [2.2 to 2.6] | 20 | 2.2 [1.8 to 2.5] |  | 0.09 [-0.3 to 0.5] | .640 |
| **Blood pressure systolic, mmHg** | | | | | | | | |
|  | Baseline | 172 | 131.6  [129.1 to 134.1] | 47 | 133.6  [128.6 to 138.6] |  |  |  |
|  | Follow-up | 74 | 133.5  [129.8 to 137.3] | 17 | 131.3  [123.9 to 138.7] |  | 4.29 [-4.3 to 12.9] | .326 |
| **Blood pressure diastolic, mmHg** | | | | | | | | |
|  | Baseline | 172 | 79.3 [78.1 to 80.6] | 47 | 78.3 [75.7 to 80.9] |  |  |  |
|  | Follow-up | 74 | 80.7 [78.7 to 82.6] | 17 | 76.9 [73.0 to 80.8] |  | 2.74 [-1,9 to 7.4] | .244 |
| **Diabetes distress score (PAID)** | | | | | | | | |
|  | Baseline | 164 | 24.8 [22.6 to 26.9] | 47 | 25.0 [20.8 to 29.1] |  |  |  |
|  | Follow-up | 129 | 23.2 [20.8 to 25.6] | 37 | 22.5 [17.9 to 27.2] |  | 0.87 [-3.9 to 5.6] | .719 |
| **Health literacy score (HLS19-Q12)** | | | | | | | | |
|  | Baseline | 164 | 33.9 [32.9 to 35.0] | 47 | 33.6 [31.6 to 35.6] |  |  |  |
|  | Follow-up | 132 | 34.9 [33.8 to 36.1] | 37 | 33.7 [31.5 to 36.0] |  | 0.87 [-1.6 to 3.3] | .482 |
